# The steady state and response to a periodic stimulation of the firing rate for a theta neuron with correlated noise

**DOI:** 10.1007/s10827-022-00836-6

**Published:** 2022-10-22

**Authors:** Jannik Franzen, Lukas Ramlow, Benjamin Lindner

**Affiliations:** 1 Department of Physics, Humboldt-Universität zu Berlin, Newtonstr. 15, Berlin, 12489 Germany; 2 Bernstein Center for Computational Neuroscience Berlin, Philippstr. 13, Haus 2, Berlin, 10115 Germany

**Keywords:** Neuron model, Spike train variability, Neural signal transmission, Stochastic neuron model

## Abstract

The stochastic activity of neurons is caused by various sources of correlated fluctuations and can be described in terms of simplified, yet biophysically grounded, integrate-and-fire models. One paradigmatic model is the quadratic integrate-and-fire model and its equivalent phase description by the theta neuron. Here we study the theta neuron model driven by a correlated Ornstein-Uhlenbeck noise and by periodic stimuli. We apply the matrix-continued-fraction method to the associated Fokker-Planck equation to develop an efficient numerical scheme to determine the stationary firing rate as well as the stimulus-induced modulation of the instantaneous firing rate. For the stationary case, we identify the conditions under which the firing rate decreases or increases by the effect of the colored noise and compare our results to existing analytical approximations for limit cases. For an additional periodic signal we demonstrate how the linear and nonlinear response terms can be computed and report resonant behavior for some of them. We extend the method to the case of two periodic signals, generally with incommensurable frequencies, and present a particular case for which a strong mixed response to both signals is observed, i.e. where the response to the sum of signals differs significantly from the sum of responses to the single signals. We provide Python code for our computational method: https://github.com/jannikfranzen/theta_neuron.

## Introduction

Neural spiking is a random process due to the presence of multiple sources of noise. This includes the quasi-random input received by a neuron which is embedded in a recurrent network (network noise), the unreliability of the synapses (synaptic noise), and the stochastic opening and closing of ion channels (channel noise) (Gabbiani & Cox, 2017; Koch, 1999). This stochasticity and the resulting response variability is a central feature of neural spiking (Holden, 1976; Tuckwell, 1989). Therefore, studies in computational neuroscience have to account for this stochasticity as it has important implication for the signal transmission properties.

Computational studies of stochastic neuron models often assume that the driving fluctuations are temporally uncorrelated. This white-noise assumption implies that the correlation time $\tau _s$ of the input fluctuations is much smaller than the time scale of the membrane potential $\tau _m$. Put differently, the input noise is regarded as fast compared to every other process present in the neural system. This assumption grants a far-reaching mathematical tractability of the problem (Abbott & van Vreeswijk, 1993; Brunel, 2000; Burkitt, 2006; Holden, 1976; Lindner & Schimansky-Geier, 2001; Ricciardi, 1977; Richardson, 2004; Tuckwell, 1989) but is violated in a number of interesting cases. First, fluctuations that arise in a recurrent network often exhibit reduced power at low frequencies (green noise) (Bair et al., 1994; Câteau & Reyes, 2006; Pena et al., 2018; Vellmer & Lindner, 2019). Second, fluctuations in oscillatory systems, e.g. caused by the electroreceptor of the paddlefish, can be band-pass filtered (Bauermeister et al., 2013; Neiman & Russell, 2001). Finally and most prominently, fluctuations that emerge due to synaptic filtering of postsynaptic potentials (Brunel & Sergi, 1998; Lindner, 2004; Lindner & Longtin, 2006 Moreno-Bote & Parga, 2010; Rudolph & Destexhe, 2005) or due to slow ion channel kinetics (Fisch et al., 2012; Schwalger et al., 2010), have reduced power at high frequencies (red noise).

There are two important types of neuron models with distinct responses characteristics: Integrators (type I neurons) and resonators (type II neurons) (Izhikevich, 2007). The canonical model for a type I neuron is the quadratic integrate-and-fire model or, mathematically equivalent, in terms of a phase variable, the theta neuron. Here we study the response characteristics of the theta neuron, driven by a low-pass filtered noise, the Ornstein-Uhlenbeck (OU) process. This model has been studied analytically by Brunel and Latham (2003) for the limits of very short and very long correlation times. Furthermore, Naundorf et al. (2005a, b) solved the associated Fokker-Planck equation for the voltage and the noise variable for selected parameter sets in order to obtain the stationary firing rate and the firing rate’s linear response to a weak periodic stimulus.

Here, we put forward semi-analytical results for the stationary firing rate by means of the matrix-continued-fraction (MCF) method for arbitrary ratios of the two relevant time scales $\tau = \tau _s/\tau _m$. We present exhaustive parameter scans of the stationary firing rate with respect to variations of the bifurcation parameter and the correlation time. Furthermore, our method also allows to calculate how a, not necessarily weak, periodic signal in the presence of a correlated background noise is encoded in the firing-rate of the model neuron. Because recently, non-weak signals, for which the linear response does not provide a good approximation to the firing rate, have attracted attention (Novikov & Gutkin, 2020; Ostojic & Brunel, 2011; Voronenko & Lindner, 2017, 2018), we also develop semi-analytical tools for the linear as well as the non-linear response of the firing rate to one or two periodic signals. To the best of our knowledge, this is the first application of the MCF method in computational neuroscience.

This paper is organized as follows. In Sect. 2 we introduce the model system and the associated Fokker-Planck equation. In Sect. 3 we compute the stationary firing rate of a theta neuron subject to correlated noise by means of the MCF method. Section 4 generalizes the ideas of the MCF method to the case where the model is driven by the OU noise and an additional periodic signal. Finally, in Sect. 4.3 we compute the firing rate response to two periodic signals. We conclude with a short summary of our results.

## Model

The quadratic integrate-and-fire (QIF) model uses the normal form of a saddle-node on invariant circle (SNIC) bifurcation (Izhikevich, 2007) with a time-dependent input $I(\hat{t})$:

1 $$\begin{aligned} \tau _m \frac{dx}{d\hat{t}} = x^2 + I(\hat{t}). \end{aligned}$$

In order to make the connection to physical time units transparent, we have kept on the l.h.s. a time constant, which is of the order of the membrane time $\tau _m$^1^, typically 10ms. In the following however for the ease of notation we use a nondimensional time $t = \hat{t}/\tau _m$, i.e. we measure time as well as any other time constants, e.g. the correlation time below, in multiples of the membrane time constant. Similarly, all frequencies and firing rates are given in multiples of the inverse membrane time constant (additional rescalings are considered below, see e.g. Eqs. (7) and (10)).

In the new nondimensional time the QIF model takes the usual form:

2 $$\begin{aligned} \frac{dx}{dt} = x^2 + I(t). \end{aligned}$$

**Fig. 1:**
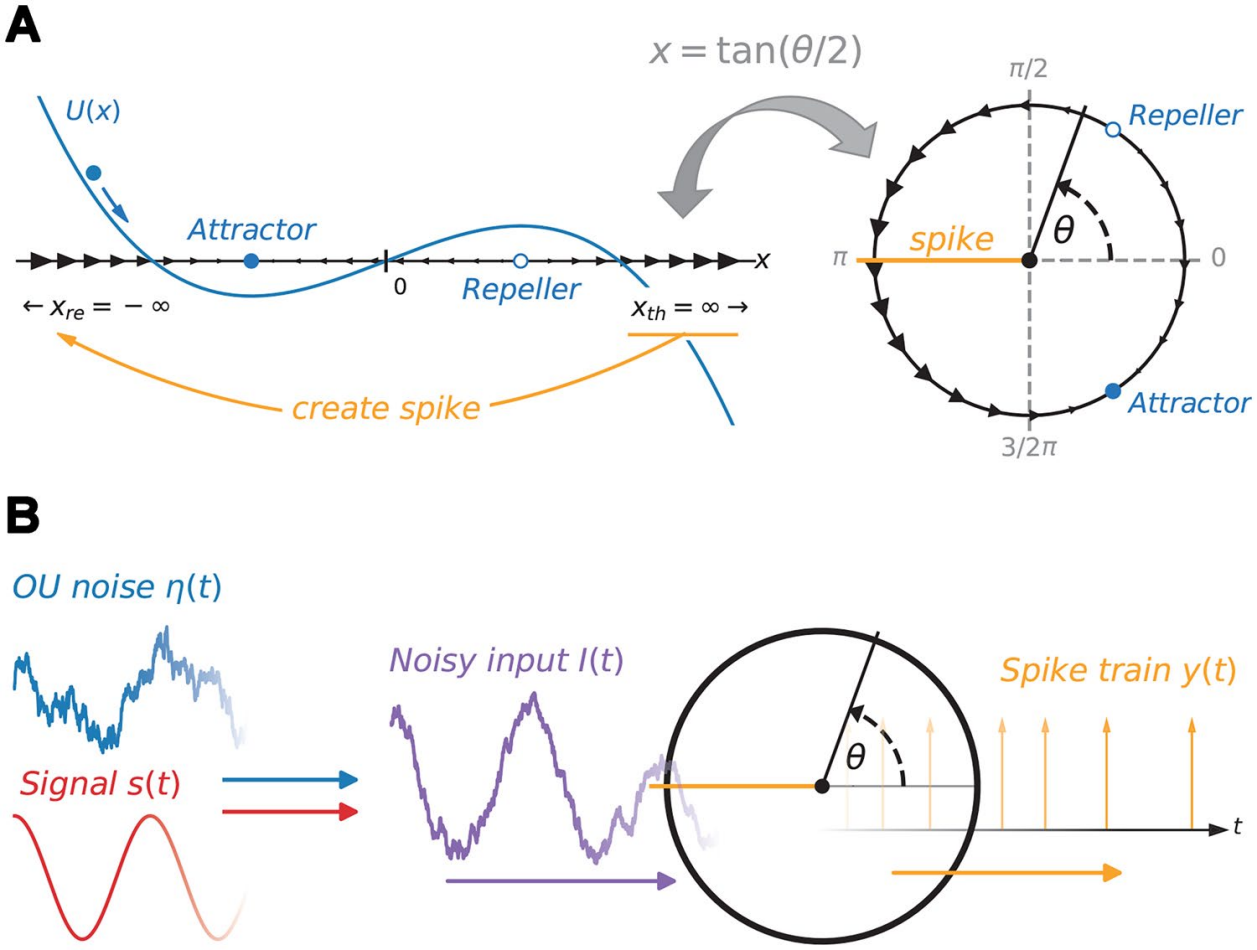
**Type-I neuron model.** **A** Representation of the deterministic QIF and the equivalent theta neuron model. The blue line shows the QIF models potential $U(x) = -\partial _x (x^2 + I)$ in the excitable regime ($I<0$). Upon reaching the threshold $x_\mathrm{th} = \infty$ a spike is created and *x* is reset to $x_\mathrm{re} = - \infty$. For the equivalent theta neuron model, obtained by the transformation $x=\tan (\theta /2)$, a spike is created whenever $\theta$ passes $\theta _\mathrm{th}=\pi$, no additional reset rule is needed. **B** Illustration of a theta neuron subject to a temporally correlated OU noise (blue) as well as a periodic signal (red) and the resulting spike train with stochastic spike times (orange)

If the variable *x*(*t*) reaches the threshold $x_\mathrm{th} = \infty$, a spike is created at time $t_i = t$ and *x*(*t*) is immediately reset to $x_\mathrm{re} = -\infty$. If the input is assumed to be constant it can serve as a bifurcation parameter and allows the model to switch between the excitable ($I<0$) and mean-driven regime ($I > 0$). The model for $I<0$ is illustrated in Fig. 1A, including the stable and unstable fixed point at $x=\pm \sqrt{I}$ as well as the reset. The QIF model can be transformed into the theta neuron by the transformation $x = \tan (\theta /2)$ (cf., Fig. 1A):

3 $$\begin{aligned} \frac{d\theta }{dt} = (1-\cos\,\theta )+(1+\cos\,\theta ) I(t). \end{aligned}$$

The advantage of such a phase description is, that the threshold $\theta _\mathrm{th} = \pi$ and reset $\theta _\mathrm{re} = -\pi$ lie at finite values. We will use this phase description of a canonical Type I neuron in the remainder of this paper.

We assume that the input *I*(*t*) consists of three parts:

4 $$\begin{aligned} I(t)&= \mu + \eta (t) + s(t), \end{aligned}$$

5 $$\begin{aligned} \tau \frac{d\eta }{dt}&= - \eta + \sqrt{2\tau \sigma ^2} \xi (t), \end{aligned}$$

a constant mean input $\mu$, a temporally correlated noise $\eta (t)$ and a periodic signal *s*(*t*) (see Fig. 1B). Note that the temporal average of the input $\bar{I} = \lim _{T\rightarrow \infty } \int _0^T I(t)dt / T$ is only affected by $\mu$ because the temporal averages are set to $\bar{\eta }(t) = 0$ and $\bar{s}(t) = 0$ without loss of generality. The correlated noise $\eta$ is given by an Ornstein-Uhlenbeck process with auto-correlation function $\langle \eta (t)\eta (t+\Delta t)\rangle = \sigma ^2 \exp (-\Delta t/\tau )$ and correlation time $\tau$; it can be generated by an extra stochastic differential equation, a trick from statistical physics known as Markovian embedding of a colored noise (see e.g. Dygas et al., 1986; Guardia et al., 1984; Langer, 1969; Mori, 1965; Siegle et al., 2010, and the review by Hänggi & Jung, 1995). We remind the reader that the correlation time $\tau$ is given in terms of the membrane time constant, i.e. $\tau = \tau _s / \tau _m$ is actually the ratio between the true correlation time $\tau _s$ (given for instance in ms) and the membrane time $\tau _m$. In the limit $\tau \rightarrow 0$ the noise $\eta (t)$ becomes uncorrelated, i.e. white. However, if the variance $\sigma ^2$ is held constant, as in Eq. (5), the effect of the noise on the neuron vanishes together with the correlation time. A non-trivial white-noise limit can be more properly described in terms of the noise intensity $D=\tau \sigma ^2$; if *D* is held constant, the noise still affects the dynamics for vanishing correlation times. For such a *constant intensity scaling* the effect of the noise vanishes as $\tau \rightarrow \infty$.

For $s(t)=0$ the system shows spontaneous spiking (not related to any signal). In this case the parameter space is three-dimensional, i.e. all statistics depend only on $(\mu ,\sigma ,\tau )$. This dependence however can be reduced to just two independent parameters ($\hat{\mu }, \hat{\tau }$) defined by

6 $$\begin{aligned} \hat{\mu } = \mu /\sigma , \qquad \hat{\tau } = \sqrt{\sigma } \tau . \end{aligned}$$

This transformation also affects the phase $\tan (\hat{\theta }/2)=\tan (\theta /2)/\sqrt{\sigma }$ and time $\hat{t}=\sqrt{\sigma }t$ in Eq. (3) and consequently rescales the firing rate

7 $$\begin{aligned} \hat{r}(\hat{t}) = r \left(\sqrt{\sigma }t\right)/\sqrt{\sigma }. \end{aligned}$$

Under an additional periodic driving $s(t) = \varepsilon \cos (\omega t)$ the signal will be rescaled as well: $\hat{s}(\hat{t}) = \hat{\varepsilon } \cos (\hat{\omega } \hat{t})$ with

8 $$\begin{aligned} \hat{\varepsilon } = \varepsilon /\sigma\,\text { and }\,\hat{\omega } = \omega /\sqrt{\sigma }. \end{aligned}$$

For several periodic signals the respective amplitudes and frequencies will be rescaled in the same manner.

For the *constant intensity scaling* we use a similar transformation and set $D=1$:

9 $$\begin{aligned} \begin{array}{l} \tilde{\mu } = \mu /D^{2/3}, \qquad \tilde{\tau } = D^{1/3} \tau ,\\ \tilde{\epsilon } = \epsilon /D^{2/3}, \qquad \tilde{\omega } = \omega /D^{1/3} \end{array} \end{aligned}$$

again, the state variables are affected by this scaling as well: $\tan (\tilde{\theta }/2)=\tan (\theta /2)/D^{1/3}$ and $\tilde{t}=D^{1/3} t$. The firing rates in the scaled and unscaled parameter space are related by

10 $$\begin{aligned} \tilde{r}(\tilde{t}) = r(D^{1/3}t)/D^{1/3}. \end{aligned}$$

We make use of these scalings in the discussion of the results. For the ease of notation, we omit the hat and tilde over the parameters.

### The Fokker-Planck equation

The stochastic system of interest can be written by two Langevin equations

11 $$\begin{aligned} \frac{d\theta }{dt}&= f(\theta , \eta , s(t)), \end{aligned}$$

12 $$\begin{aligned} \tau \frac{d\eta }{dt}&= - \eta + \sqrt{2\tau \sigma ^2} \xi (t), \end{aligned}$$

where $f(\theta , \eta , s(t)) = (1-\cos\,\theta )$ $+(1+\cos\,\theta )$ $(\mu + \eta (t) + s(t))$. The relation to the governing equation for the probability density function (PDF) is the well known Fokker-Planck equation (FPE) (Risken, 1984). The PDF denotes the probability to find the phase $\theta$ and noise $\eta$ at time *t* around certain values. In the neural context the PDF can be related to the instantaneous firing rate *r*(*t*) (see for instance Brunel & Sergi, 1998; Naundorf et al., 2005a, b, and Moreno-Bote & Parga, 2010) as we recall in the following. The FPE is given by:

13 $$\begin{aligned} \partial _t P(\theta ,\eta ,t)&= \hat{L}(\theta ,\eta , s(t)) P(\theta ,\eta ,t) \end{aligned}$$

14 $$\begin{aligned} \hat{L}(\theta ,\eta , s(t))&= - \partial _\theta \left[\,f(\theta ,\eta , s(t))\right] + \frac{1}{\tau } \partial _\eta \left[ \eta + \sigma ^2 \partial _\eta \right] . \end{aligned}$$

The two dimensional partial differential equation is completed by two natural boundary conditions

15 $$\begin{aligned} P(\theta , \eta = \infty ,t) = P(\theta , \eta = -\infty ,t) = 0, \end{aligned}$$

a periodic boundary condition

16 $$\begin{aligned} P(\theta = \pi , \eta ,t) = P(\theta = -\pi , \eta ,t), \end{aligned}$$

and the normalization condition

17 $$\begin{aligned} \int _{-\infty }^{\infty } d\eta \, \int _{-\pi }^{\pi } d\theta \, P(\theta ,\eta ,t) = 1. \end{aligned}$$

There is a corresponding continuity equation that relates the temporal derivative of the PDF to the spatial derivative of the probability current:

18 $$\begin{aligned} \partial _t P(\theta ,\eta ,t)&= -\partial _\theta J_\theta (\theta ,\eta ,t) -\partial _\eta J_\eta (\theta ,\eta ,t), \end{aligned}$$

where $J_\theta$ and $J_\eta$ are the probability currents in the $\theta$ and $\eta$ direction, respectively:

19 $$\begin{aligned} J_\theta&= f(\theta ,\eta , s(t)) P(\theta ,\eta ,t), \end{aligned}$$

20 $$\begin{aligned} J_\eta&= -\frac{1}{\tau } \left( \eta + \sigma ^2 \partial _\eta \right) P(\theta ,\eta ,t). \end{aligned}$$

An important insight is that the probability current in the phase direction $J_\theta$ at the threshold $\theta = \pi$ is directly related to the instantaneous firing rate *r*(*t*):

21 $$\begin{aligned} r(t) = \int _{-\infty }^{\infty } d\eta \, J_\theta (\pi ,\eta ,t) = 2 \int _{-\infty }^{\infty } d\eta \, P(\pi ,\eta ,t). \end{aligned}$$

In the last equality we have used that the dynamics of the theta neuron becomes independent of the input at the threshold; specifically, we have $f(\pi ,\eta , s(t)) = 2$.

The solution of the two-dimensional Fokker-Planck equation and the boundary conditions listed above is a difficult problem, even in the simplest case of the (time-independent) stationary solution in the absence of a periodic stimulus. Different authors have proposed approximate solutions in limit cases, e.g. for the case of very slow or very fast Ornstein-Uhlenbeck noise (Brunel & Latham, 2003), for weak noise in the mean-driven regime (Galán, 2009; Zhou et al., 2013), or, in the case of a periodic modulation of the firing rate, for very low or very high stimulus frequencies (Fourcaud-Trocmé et al., 2003). A numerical method to solve the two-dimensional Fokker-Planck equation in terms of an eigenfunction expansion was presented by Naundorf et al. (2005a, b); similar approaches have been pursued to describe two one-dimensional white-noise driven neuron models either coupled directly (Ly & Ermentrout, 2009) or subject to a shared input noise (Deniz & Rotter, 2017). Eigenfunction expansions have also been used to describe the activity in neural populations and neural networks, see e.g. Knight (2000) and Doiron et al. (2006). Turning back to the problem of single-neuron models, beyond the theta neuron, different approximations to the multi-dimensional Fokker-Planck equation for neuron models with Ornstein-Uhlenbeck noise have been suggested for the perfect integrate-and-fire model (Fourcaud & Brunel, 2002; Lindner, 2004; Schwalger et al., 2010, 2015) and for the leaky integrate-and-fire model (Alijani & Richardson, 2011; Brunel & Sergi, 1998; Brunel et al., 2001; Moreno et al., 2002; Moreno-Bote & Parga, 2004, 2006, 2010; Schuecker et al., 2015; Schwalger & Schimansky-Geier, 2008). We note that with respect to the driving noise, the related simpler case of an exponentially correlated two-state (dichotomous) noise permits the exact analytical solution for a few statistical measures such as the firing rate and stationary voltage distribution (Droste & Lindner, 2014; Müller-Hansen et al., 2015), the power spectrum and linear response function (Droste & Lindner, 2017), and the serial correlation coefficient of the interspike intervals (Lindner, 2004; Müller-Hansen et al., 2015).

## Stationary firing rate

If we consider a system that is subject to a temporally correlated noise but no external signal ($s(t) = 0$) then the probability density asymptotically approaches a stationary distribution $P_0(\theta , \eta )$ which is what we consider now. The FPE for this stationary distribution reads

22 $$\begin{aligned} 0 = \hat{L}_0(\theta ,\eta ) P_0(\theta ,\eta ), \end{aligned}$$

with the stationary Fokker-Planck operator $\hat{L}_0(\theta ,\eta ) = \hat{L}(\theta , \eta , 0)$. Once the stationary probability density is known it can be used to obtain the stationary firing rate $r_0$. Alternatively to Eq. (21) one can calculate the firing rate by

23 $$\begin{aligned} 2\pi r_0 = \int _{-\pi }^{\pi } d\theta \, \int _{-\infty }^{\infty } d\eta \, J_{\theta ,0}(\theta ,\eta ), \end{aligned}$$

where $J_{\theta ,0}(\theta ,\eta )$ denotes the component of the probability current in the direction of the phase for $s(t)\equiv 0$. To see how to arrive at this equation, we take the stationary case of Eq. (18) and integrate it over all values of $\eta$. The integral term $\int d\eta \, \partial _\eta J_\eta = J(\theta , \eta = \infty ) - J(\theta , \eta = -\infty )$ vanishes because of the natural boundary conditions and it follows that $\partial _\theta \int d\eta \, J_\theta = 0$. Consequently the integrated $\theta$ current does not depend on $\theta$ and is everywhere equal to the firing rate. An additional integration over $\theta$, yielding the factor $2\pi$, leads to Eq. (23).

**Fig. 2:**
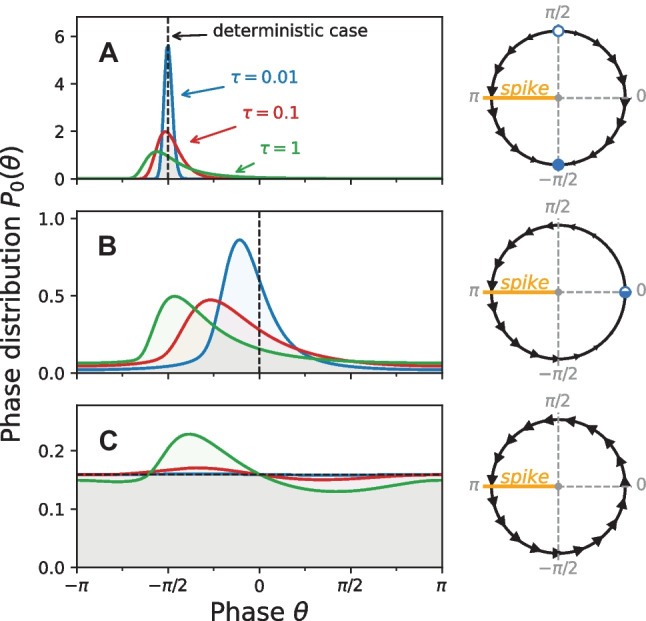
**Stationary phase distribution** of the theta neuron in the excitable regime ($\mu =-1$, **A**), at the bifurcation point ($\mu =0$, **B**) and in the mean-driven regime ($\mu =1$, **C**). Dynamics of the corresponding deterministic systems are shown at the right. For the phase distributions the variance of the OU noise is held constant at $\sigma ^2=1$ while the correlation time varies as shown in A. For $\tau \rightarrow 0$ the effect of the noise vanishes, i.e. the model becomes deterministic. The distributions have been calculated using the MCF method. Parameters MCF method: $n_\text {max} = p_\text {max} = 200$

**Fig. 3:**
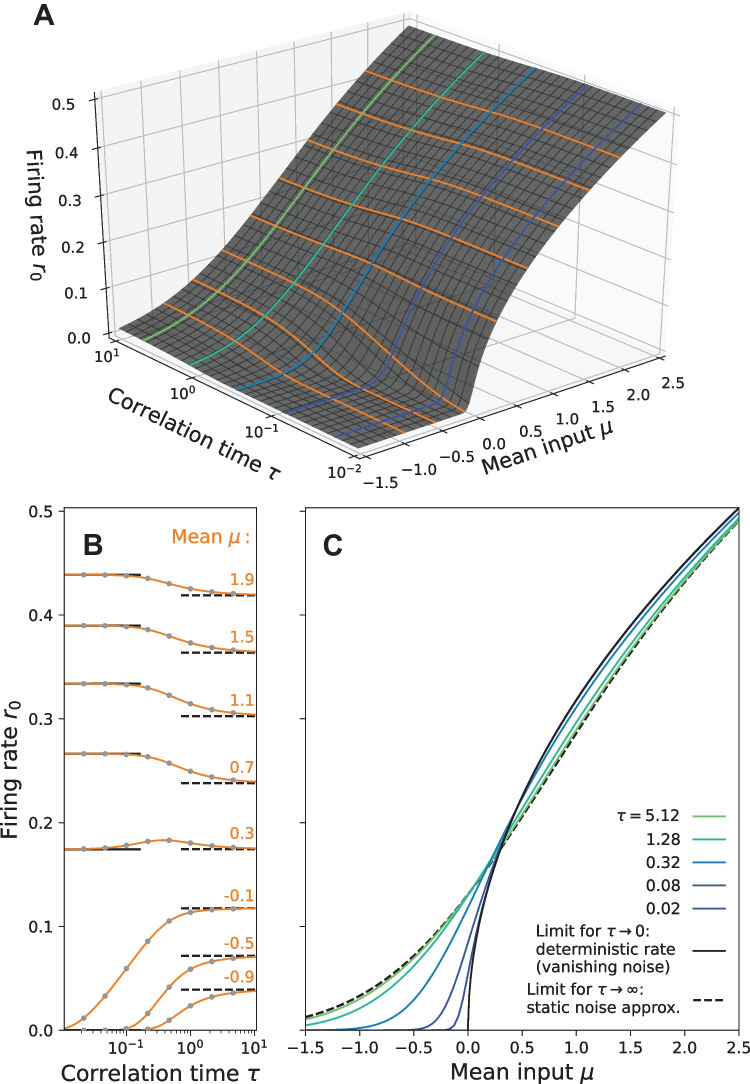
**Stationary firing rate** in the constant variance scaling ($\sigma ^2=1$) for different values of $\mu$ and $\tau$. Contour lines from **A** are shown again in **B** and **C**. Interestingly, the firing rate of the theta neuron can increase, decrease and even exhibit non-monotonic behavior with respect to the correlation time $\tau$ of the OU noise as shown in B. Calculations by the MCF Method are confirmed by stochastic simulations (gray dots). Parameters MCF method: $n_\text {max} = p_\text {max} = 150$

### The MCF method

In the previous section it was shown that the stationary probability density is interesting on its own because it is directly related to the stationary firing rate. Here we outline the core ideas and assumptions that are necessary to compute the stationary PDF $P_0(\theta ,\eta )$ by means of the matrix-continued-fraction method, which has been put forward by Risken (1984).

As a first step, the stationary probability density is expanded with respect to the phase $\theta$ and noise $\eta$ by two sets of eigenfunctions, namely the complex exponential functions $e^{i n \theta }/\sqrt{2\pi }$ and Hermite functions $\phi _p(\eta )$ (see Bartussek, 1997 for a similar choice):

24 $$\begin{aligned} P_0(\theta ,\eta ) = \frac{\phi _0(\eta )}{2 \pi } \sum _{p=0}^{\infty } \sum _{n=-\infty }^{\infty } c_{n,p} e^{i n \theta } \phi _p(\eta ). \end{aligned}$$

Note, that $\left( c_{n,p}\right) ^* = c_{-n,p}$ because $P_0(\theta , \eta )$ is real. Thus, we must only determine the expansion coefficients for $n \ge 0$. Both sets satisfy the periodic and natural boundary conditions in $\theta$ and $\eta$, respectively. A first application of this result is the determination of the marginal probability density by

25 $$\begin{aligned} P_0(\theta ) := \int _{-\infty }^\infty d\eta \, P_0(\theta ,\eta ) = \frac{1}{2 \pi } \sum _{n=-\infty }^{\infty } c_{n,0} e^{i n \theta } \end{aligned}$$

which is illustrated for different values of $\mu$ and $\tau$ in Fig. 2. The stationary firing rate is conveniently expressed by only two of the coefficients,

26 $$\begin{aligned} r_0=\frac{(1+\mu ) - (1-\mu )\text {Re}(c_{1,0}) + \sigma \text {Re}(c_{1,1})}{2\pi }. \end{aligned}$$

This expression can be derived by inserting the expansion into Eq. (23) and using the properties of the coefficients and eigenfunctions, in particular (80) and (81) of the Hermite functions.

The coefficients can be determined by a substitution of the expansion Eq. (24) into the stationary FPE (22) which yields the tridiagonal recurrence relation, see Appendix A:

27 $$\begin{aligned} \hat{K}_n \varvec{c}_n = \varvec{c}_{n-1} + \varvec{c}_{n+1} \end{aligned}$$

with the coefficient vectors $\varvec{c}_n = \left( c_{n,0}, c_{n,1}, ... \right) ^T$ and $\varvec{c}_0 = \left( 1,0,0, ... \right) ^T$. The matrix $\hat{K}_n$ is given by

28 $$\begin{aligned} \hat{K}_n&= 2 \left( \hat{B}^{-1}-\mathbbm {1} \right) - \frac{\hat{B}^{-1} \hat{A}}{n}, \end{aligned}$$

where $\mathbbm{1}$ is the identity matrix and $\hat{A}$, $\hat{B}$ are defined by

29 $$\begin{aligned} \left( \hat{A} \right) _{p,q}&= i \frac{q}{\tau _s} \delta _{p,q}, \end{aligned}$$

30 $$\begin{aligned} \left( \hat{B} \right) _{p,q}&= \frac{1-\mu }{2} \delta _{p,q} - \frac{\sigma }{2} \left( \sqrt{q} \delta _{p+1,q} + \sqrt{q+1} \delta _{p-1,q} \right) . \end{aligned}$$

Solving Eq. (27) for $c_{n,p}$ is difficult because the matrices are infinite and the equation constitutes a relation between *three* unknown. As a first step to find the coefficients, one can truncate the expansion in Eq. 24 to obtain finite matrices. In practice, we assume that all Hermite functions and Fourier modes become negligible for large *p* or *n*, so that the corresponding coefficients vanish^2^$c_{n,p} = 0$ for $p>p_\text {max}$ or $n>n_\text {max}$. To solve the second problem (of having three unknowns), we define transition matrices $\hat{S}_n$ by

31 $$\begin{aligned} \varvec{c}_{n+1} = \hat{S}_n \varvec{c}_n, \end{aligned}$$

which upon insertion into Eq. (27) yield:

32 $$\begin{aligned} 0 = \left[ \left( \hat{K}_n-\hat{S}_n \right) \hat{S}_{n-1} - \mathbbm {1} \right] \varvec{c}_{n-1}. \end{aligned}$$

For any coefficient vectors $\varvec{c}_n$ this equation is satisfied provided the term in square brackets vanishes. The relation between the two unknown transition matrices can be expressed by:

33 $$\begin{aligned} \hat{S}_{n-1} = \left[ \hat{K}_n -\hat{S}_n \right] ^{-1} \end{aligned}$$

and leads by recursive insertion to an infinite matrix continued fraction

34 $$\begin{aligned} \hat{S}_{n} = \frac{1}{\hat{K}_n -\frac{\displaystyle 1}{\hat{K}_{n+1} -\frac{1}{...} } }, \end{aligned}$$

where $1/\cdot$ denotes the inverse of a matrix. This fraction is truncated after $n>n_\mathrm{max}$. The matrix $\hat{S}_0$ determines the following coefficients via Eq. (31):

35 $$\begin{aligned} c_{1,0} = \left( \hat{S}_0\right) _{0,0};\quad c_{1,1} = \left( \hat{S}_0\right) _{1,0}, \end{aligned}$$

which are needed for the computation of the firing rate according to Eq. (26).

### Constant variance scaling

The MCF method provides a fast computational method to determine the stationary firing rate $r_0$ in a large part of the parameter space. Together with different analytical approximations it is possible to cover the complete dependence of $r_0$ on the parameters $\mu$, $\tau$ and $\sigma$. In the following figures, we additionally verify the MCF results by comparison to numerical simulations of Eq. (11) using a Euler-Maruyama scheme with time step $\Delta t = 5 \cdot 10^{-3}$ for $N_\text {trials} = 5 \cdot 10^5$ trials of length $T_\text {max} = 500$. For more details see the repository. In Fig. 3 we use the constant variance scaling (see Sect. 2) with $\sigma =1$. A different choice for $\sigma$ would result in a rescaling of the axes according to Eq. (6). As depicted in Fig. 3B, for short as well as large correlation times, the firing rate approaches limit values indicated by the horizontal lines. For $\tau \rightarrow 0$, the effect of the correlated noise vanishes so that the short time limit is equal to the deterministic firing rate

36 $$\begin{aligned} r_{\text {det}} = r(\mu , \tau =0) = \frac{\sqrt{\mu }}{\pi }\Theta (\mu ), \end{aligned}$$

where $\Theta (\mu )$ is the Heaviside function. In the case $\tau \rightarrow \infty$, the noise causes a slow modulation of the firing rate; computing the long-correlation-time limit then corresponds to averaging the deterministic firing rate over the distribution of the noise (quasi-static noise approximation, see Moreno-Bote & Parga, 2010)

37 $$\begin{aligned} r_\infty (\mu ) = \int _{-\infty }^{\infty } dI \,  {P}_\eta (I-\mu ) r_\text {det} (I). \end{aligned}$$

**Fig. 4:**
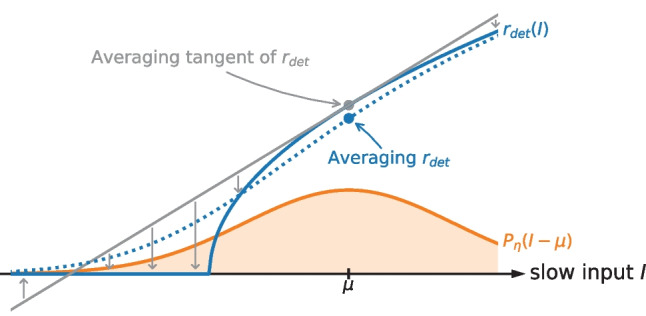
**Mechanism for the firing rate reduction.** The decrease of the firing rate due to strongly correlated noise in the mean-driven regime is a consequence of the concave curvature of the deterministic firing rate $r_\text {det}(I)$. For large $\tau$ the firing rate can be approximated by averaging the deterministic firing rate over the noise distribution according to Eq. (37), this yields the blue point on the dashed line

We recall that for a QIF model driven by white noise the firing rate is always larger than the deterministic rate (Lindner et al., 2003). In contrast, a colored noise may decrease the firing rate (Brunel & Latham, 2003; Galán, 2009) as shown in Fig. 3. For large correlation times, the decrease in the firing rate is a direct consequence of the concave curvature of the deterministic firing rate $r_\text {det}(I)$ at large $\mu$ as illustrated in Fig. 4. This can be understood as follows. If we take the linear approximation of the deterministic rate around the operation point $\mu$ then, not surprisingly, with a symmetric input distribution of the noise, the averaging yields the deterministic firing rate at the operation point:

38 $$\begin{aligned} \int dI P_\eta(I-\mu ) \underline{\left( \left. \frac{dr_\text {det}}{dI}\right| _{I=\mu } (I-\mu ) + r_\text {det}(\mu )\right) } = r_\text {det} . \end{aligned}$$

In the relevant range the underlined term is larger than the function $r_\text {det}(I)$ in Eq. (37) as it can be seen from Fig. 4. Consequently, the resulting integral in Eq. (38) (i.e. the deterministic firing rate) is larger than the actual firing rate in the long-correlation-time limit, Eq. (37). This is the mechanism by which a colored noise can reduce the firing rate in the mean-driven regime.

**Fig. 5:**
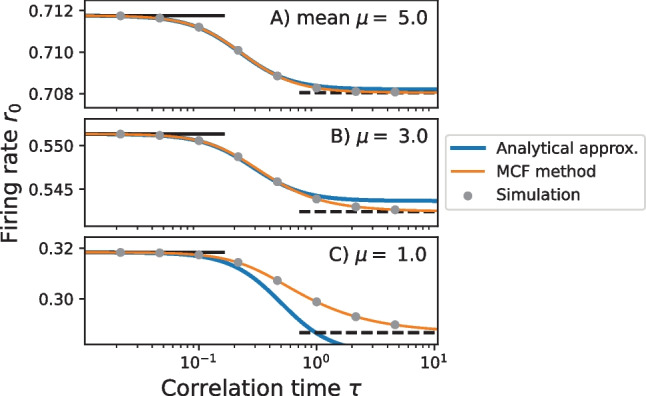
**Decrease of the firing rate** with respect to the correlation time $\tau$ at fixed variance $\sigma ^2=1$. Analytical approximations according to Eq. (39) (blue line) are compared to the firing rate obtained by the MCF method (orange line) and again verified by stochastic simulations (gray dots). Parameters MCF method: $n_\text {max} = p_\text {max} = 200$

For weak noise in the mean-driven regime ($\sigma \ll \mu$) this drop in the firing rate can be calculated analytically as done by Galán (2009). The formula requires the phase response curve (PRC) of the theta neuron, which is well known (Ermentrout, 1996), resulting in the following compact expression for the firing rate:

39 $$\begin{aligned} r_0 \approx r_{\text {det}} - \frac{\sigma ^2}{2 \pi } \frac{\tau ^2 /\sqrt{\mu }}{4\mu \tau ^2+1} \end{aligned}$$

(please note the transition from cyclic frequencies used in Galán, 2009 to firing rates). The formula predicts clearly a reduction of the firing rate by colored noise; specifically, $r_0$ decreases monotonically with increasing correlation times. It should be noted, however, that in the strongly mean-driven regime, in which this theory is valid, the changes in the firing rate are very small (see Fig. 5A, B). If the driving is less strong and deviations of the firing rate from $r_\text {det}$ are more pronounced, the theory according to Eq. (39) no longer provides a good approximation, (see Fig. 5C).

**Fig. 6:**
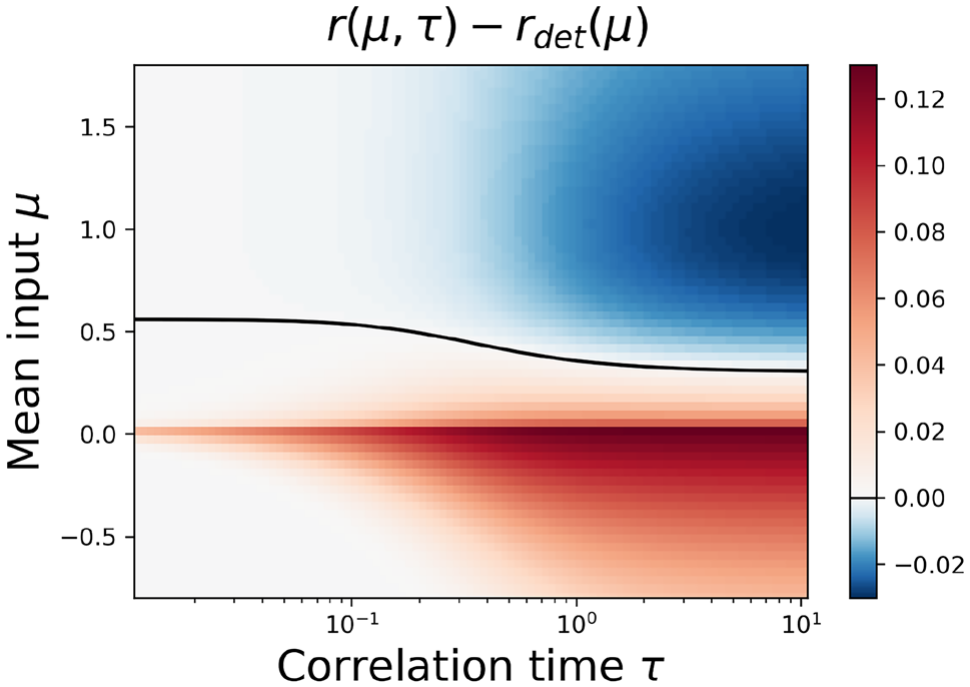
**Comparison between the firing rate and the deterministic rate.** Difference between $r(\mu ,\tau )$ and the deterministic firing rate $r_\mathrm{det}(\mu )$ for $\sigma ^2 = 1$. As expected, in the excitable regime ($\mu < 0$) the firing rate of the stochastic system is increased compared to the deterministic rate. For the mean-driven regime ($\mu > 0$) the firing rate can be both increase or decreased depending on the particular value of both $\mu$ and $\tau$. Parameters MCF method: $n_\text {max} = p_\text {max} = 150$

Is at least the qualitative prediction of an overall rate reduction due to correlated noise correct? To answer this question, we plot in Fig. 6 the difference between the firing rate and the deterministic limit $r_0-r_\text {det}$ for a broad of correlation times $\tau$ and inputs $\mu$. This difference can be both positive and negative. Trivially, in the excitable regime ($\mu <0$) the firing rate in the presence can only be larger than the vanishing deterministic rate (here $r_\text {det} = 0$). In the mean-driven regime the changes can be both positive (for sufficiently small $\mu$) and negative (for larger $\mu$); the exact line of separation is displayed by a solid line in Fig. 6.

### Constant intensity scaling

**Fig. 7:**
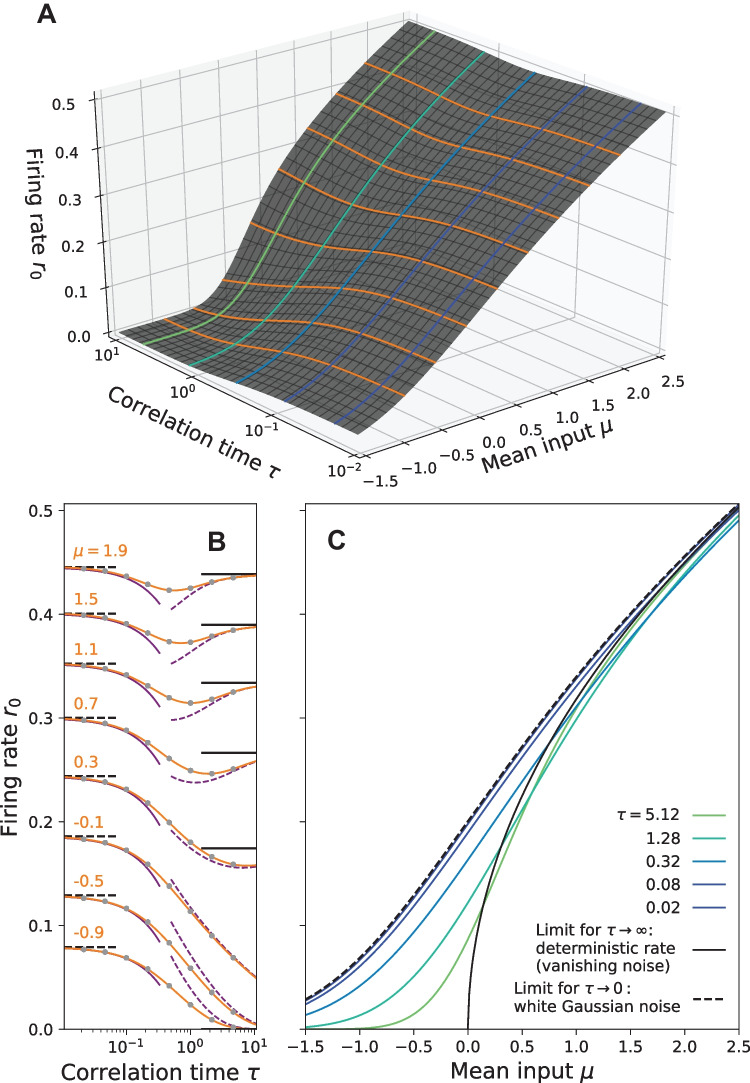
**Stationary firing rate** in the constant intensity scaling ($D=1$) for different values of $\mu$ and $\tau$. Contour lines from **A** are shown again in **B** and **C**. Interestingly, the firing rate is always smaller than the corresponding white noise limit $\tau \rightarrow 0$ (dashed line) and can show non-monotonic behavior with a minimum depending on $\tau$ and $\mu$, see B. Here, known analytical approximations by Fourcaud-Trocmé et al. (2003) (solid purple lines) and Moreno-Bote and Parga (2010) (dashed purple lines) are compared to calculations by the MCF Method (orange lines). Parameters MCF method: $n_\text {max} = p_\text {max} = 150$

Instead of a constant variance, we can also keep the noise intensity fixed ($D=\sigma ^2 \tau$). The corresponding stationary firing rate as a function of $\mu$ and $\tau$ is shown in Fig. 7A. One advantage of the constant-intensity scaling is that it permits a non-trivial white noise limit ($\tau \rightarrow 0$), displayed in Fig. 7B, C by the dashed lines (Brunel & Latham, 2003). In the opposite limit of a long correlation time the noise variance vanishes, which implies that $r_0$ approaches the deterministic rate.

Remarkably, for a sufficiently strong mean input current $\mu$, the rate attains a minimum at intermediate correlation times. Considering the long as well as the short correlation-time approximation by Moreno-Bote and Parga (2010) (see our Eq. (37)) and Brunel and Latham (2003) (see Eq. (3.19) therein), respectively, this behavior can be expected. Generally, we find that the firing rate for any $\tau$ is smaller than the white-noise limit.

## Response to periodic stimulus

**Fig. 8:**
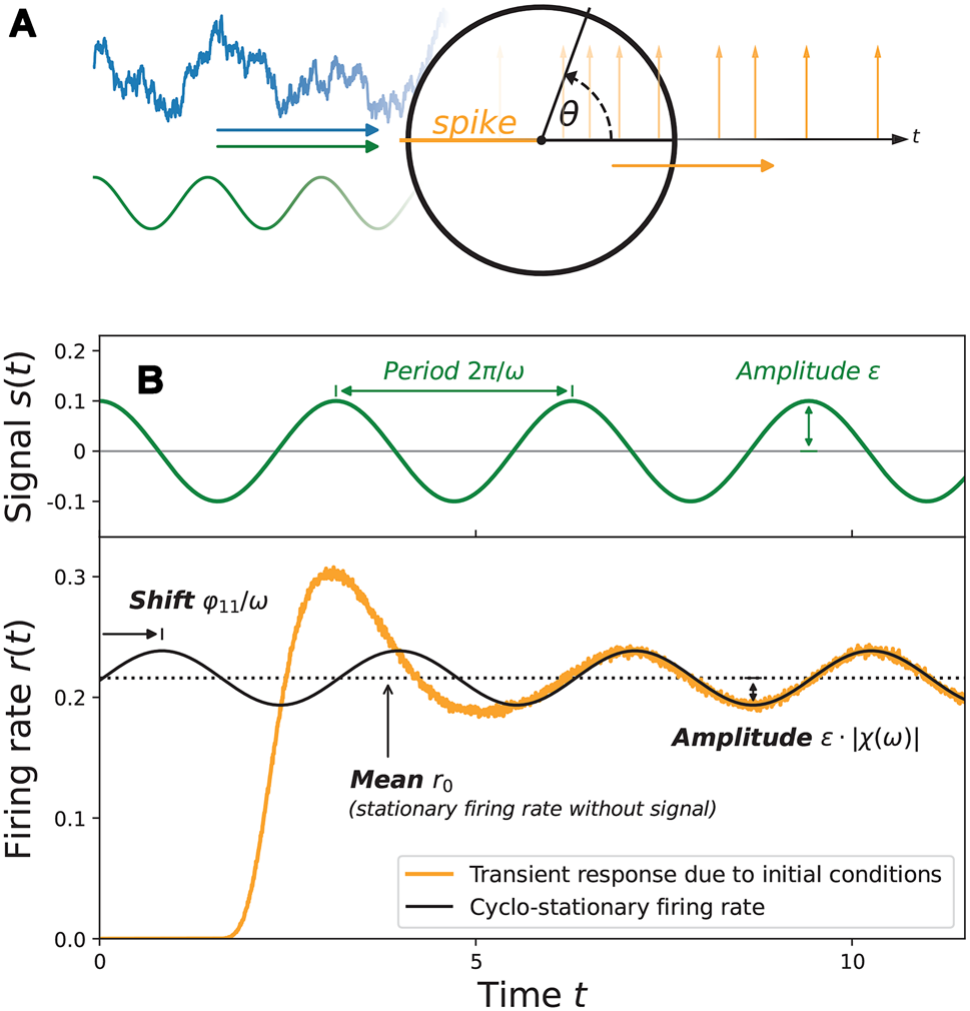
**Cyclo-stationary firing rate.** **A** Illustration of a theta neuron model subject to a temporally correlated OU noise and a periodic signal. **B** The firing rate (orange line; simulation) approaches a cyclo-stationary state (black line; MCF method) due to the periodicity of the signal (green line). In the linear regime the firing rate is well approximated by $r(t) \approx r_0 + \vert \chi (\omega )\vert s(t-\varphi _{11}/\omega )$. Parameters: $\mu = 0.5$, $\sigma ^2 = 1$, $\tau = 1$, $\varepsilon = 0.1$, and $\omega = 2$. The cyclo-stationary firing rate was calculated by the MCF method with $n_\text {max} = p_\text {max} = 100$. Simulation parameters: In this figure, the number of realizations was up-scaled to $N_\text {trials} = 1 \cdot 10^6$ for visual purposes. For all realizations, the initial values are $\eta (t=0) = 0$ and $\theta (t=0) = -\pi$

In the previous section we have considered a theta neuron with an input current *I*(*t*) that consisted of a constant input $\mu$ and a colored noise $\eta (t)$. We now turn to a more general case that involves an additional periodic signal

40 $$\begin{aligned} s(t) = \varepsilon \cos \left( \omega t \right) \end{aligned}$$

as illustrated in Fig. 8A and demonstrate how the MCF method can be used to compute the response of the firing rate.

We consider the time-dependent signal *s*(*t*) as a perturbation with amplitude $\varepsilon$. The respective FPE can be expressed by the stationary Fokker-Planck operator $\hat{L}_0$ as defined in the last section and an additional term that represents the effect of the periodic signal:

41 $$\begin{aligned} \partial _t P(\theta ,\eta ,t) = \left( \hat{L}_0(\theta ,\eta ) - s(t) \hat{L}_\text {per}\right) P(\theta ,\eta ,t) \end{aligned}$$

with $\hat{L}_\text {per} = \partial _\theta (1+\cos \theta )$. As a result of the periodic forcing, we can no longer expect that the probability density converges to a stationary distribution; instead the probability density approaches a so called cyclo-stationary state with period $T = 2\pi / \omega$:

42 $$\begin{aligned} P(\theta ,\eta ,t+T) = P(\theta ,\eta ,t). \end{aligned}$$

Since this distribution fully determines the asymptotic firing rate, this implies for the latter $r(t + T) = r(t)$.

To determine the cyclo-stationary PDF we again use a twofold expansion, first a Fourier expansion that reflects the periodic nature of the signal and second a Taylor expansion with respect to the small amplitude of the periodic signal $\varepsilon$:

43 $$\begin{aligned} P(\theta ,\eta ,t)&= \sum _{\ell =0}^{\infty } \sum _{k=-\infty }^{\infty } \varepsilon ^\ell e^{-i k \omega t} P_{\ell , k} (\theta ,\eta ). \end{aligned}$$

Note that $P_{\ell , k}(\theta , \eta )=P^*_{\ell , -k}(\theta , \eta )$ because $P(\theta , \eta , t)$ is real. The expansion Eq. (43) can be substituted into Eq. (41) to obtain a system of coupled differential equations that are no longer time dependent and can be solved iteratively with respect to $\ell$:

44 $$\begin{aligned} \hat{L}_k P_{\ell , k} = {\left\{ \begin{array}{ll} 0 &{} \ell = 0, \\ \frac{1}{2}\hat{L}_\text {per}(P_{\ell -1, k-1} + P_{\ell -1, k+1}) &{} \ell > 0, \end{array}\right. } \end{aligned}$$

with $\hat{L}_k = \hat{L}_0 + ik\omega$. The normalization of the probability density provides additional conditions for these functions:

45 $$\begin{aligned} \int _{-\pi }^{\pi } d\theta \int _{-\infty }^{\infty } d\eta \, P_{\ell , k} (\theta ,\eta ) = \delta _{k, 0}\delta _{\ell ,0}. \end{aligned}$$

Here $\delta _{i,j}$ is the Kronecker delta. Clearly, $P_{0,0}(\theta , \eta ) = P_0(\theta , \eta )$ is the stationary probability density. This system of coupled differential equations Eq. (44) can be solved iteratively ($\ell \rightarrow \ell +1$). Notice that whenever $P_{\ell , k}(\theta , \eta )$ is governed by a homogeneous differential equation, i.e. $\hat{L}_k P_{\ell , k}(\theta , \eta ) = 0$, the trivial solution $P_{\ell , k}(\theta , \eta ) = 0$ does satisfy Eq. (45) and is thus a solution (except for $k=\ell =0$). Therefore, for $\ell =0$ we find that all coefficients except $P_{0,0}(\theta , \eta )$ vanish. For $\ell =1$ we find two non-vanishing coefficients, namely $P_{1,-1}(\theta , \eta )$ and $P_{1,1}(\theta , \eta )$. Generally, all coefficients $P_{\ell ,k}(\theta , \eta )$ for $k>\ell$ and $k + \ell = \text {odd}$ vanish (see Fig. 16). The remaining inhomogeneous differential equations can be solved by means of the MCF method (see Appendix B).

The cyclo-stationary firing rate can now be expressed in terms of the functions $P_{\ell , k}(\theta , \eta )$ using Eq. (21), exploiting the symmetry $P_{\ell , k} =P^*_{\ell , -k}$ and $P_{\ell , k>\ell }=0$:

46 $$\begin{aligned} r(t) = \sum _{\ell =0}^{\infty } \sum _{k=0}^{\ell } \varepsilon ^\ell \vert r_{\ell , k}(\omega )\vert \cos (k \omega t - \varphi _{\ell , k}(\omega )), \end{aligned}$$

with:

47 $$\begin{aligned} r_{\ell , k} = 2(2 - \delta _{k,0}) \int \limits _{-\infty }^\infty d\eta \, P_{\ell , k}(\pi ,\eta ), \;\; \varphi _{\ell , k} = \arg (r_{\ell , k}), \end{aligned}$$

where arg($\cdot$) is the complex argument. We recover our well known stationary firing rate for $\ell =k=0$, i.e. $r_{0,0} = r_0$. Note that some of the terms $r_{\ell , k}$ in Eq. (46) vanish because of the underlying symmetry of the governing equations Eq. (44).

### Linear response

For small $\varepsilon$ the linear term in the expansion, i.e. the linear response $r_{1,1}$, already provides a good approximation of the asymptotic firing rate *r*(*t*):

48 $$\begin{aligned} r(t) \approx r_0 + \varepsilon \vert r_{1,1}(\omega )\vert \cos (\omega t - \varphi _{1,1}). \end{aligned}$$

Note that all other terms $r_{1, k\ne 1}$ vanish. The function $\vert r_{1,1}(\omega ) \vert$ is also commonly known as the absolute value of the susceptibility $\vert \chi (\omega ) \vert$ that quantifies the amplitude response of the firing rate. The phase shift with respect to the signal is described by $\varphi _{1,1}$. An exemplary signal *s*(*t*) together with the linear response, given in terms of the amplitude and phase shift, is shown in Fig. 8. For the chosen small signal amplitude $\varepsilon$, the linear theory indeed captures very well the cyclo-stationary part of the firing rate. There is also a transient response due to the chosen initial condition of the ensemble, here we however focus solely on the cyclo-stationary response.

**Fig. 9:**
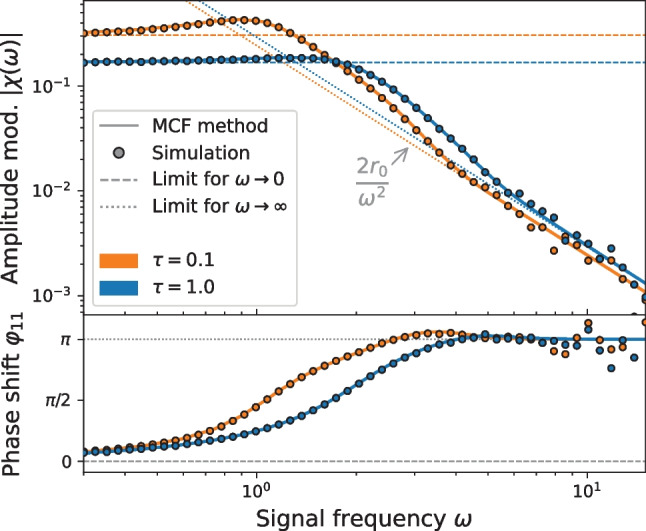
**Susceptibility and phase shift.** The absolute value of the susceptibility $\vert \chi (\omega )\vert$ and phase shift $\varphi _{11}$ are computed by the MCF method for two different correlation times $\tau$. The results are confirmed by stochastic simulations and compared to known limit cases for $\omega \rightarrow 0$ and $\omega \rightarrow \infty$ according Eqs. (50) and (51), respectively. Parameters: $\mu =0.1$, $\sigma ^2 = 1$. Parameters MCF method: $n_\text {max} = p_\text {max} = 200$. Simulation parameters: $T=5 \cdot 10^3$, $dt = 1 \cdot 10^{-2}$ and $N_\text {trials} = 1.6 \cdot 10^4$

Before we discuss the rate modulation with respect to different parameters, we compare our numerical results against known approximations (Fourcaud-Trocmé et al., 2003) (see Fig. 9). First we verify the low frequency limit $\omega \rightarrow 0$. In this case the signal *s*(*t*) is slow and can be considered as a quasi-constant input. Expanding the firing rate with respect to the signal amplitude $\varepsilon$ yields:

49 $$\begin{aligned} r(t) = r_0(\mu +s(t)) \approx r_0 + \frac{\partial r_0}{\partial \mu } s(t). \end{aligned}$$

A comparison with Eq. (48) allows to identify the low frequency limit of the susceptibility and phase shift:

50 $$\begin{aligned} \vert \chi (\omega \rightarrow 0)\vert&= \frac{\partial r_0}{\partial \mu },&\varphi _{1,1}(\omega \rightarrow 0)&=0. \end{aligned}$$

As we can compute the firing rate $r_0$ for different values of $\mu$ (see Sect. 3), the derivative above can be calculated numerically.

Second, in the opposite limit of large frequencies $\omega \rightarrow \infty$, the theta neuron acts as a low-pass filter (Fourcaud-Trocmé et al., 2003):

51 $$\begin{aligned} \vert \chi (\omega \rightarrow \infty )\vert&= \frac{2 r_0}{\omega ^2},&\varphi (\omega \rightarrow \infty )&= \pi \end{aligned}.$$

Hence, the susceptibility becomes very small in the high-frequency limit which is also noticeable by the pronounced random deviations of our simulation results in this specific limit. Both limit cases are well captured by our method for two values of the correlation time ($\tau =0.1, 1$) in the mean driven regime. We see that here the main effect of increasing the correlation time is to diminish the resonance of the response: For $\tau =0.1$ the susceptibility peaks around $\omega \approx 2\pi r_0$ (note, that for small $\tau$: $r_0 \approx r_\text {det}$); this peak is gone for $\tau =1$ because the effect of the noise, keeping its variance constant, increases with $\tau$. All these features are in detail confirmed by the results of stochastic simulations (symbols in Fig. 9).

**Fig. 10:**
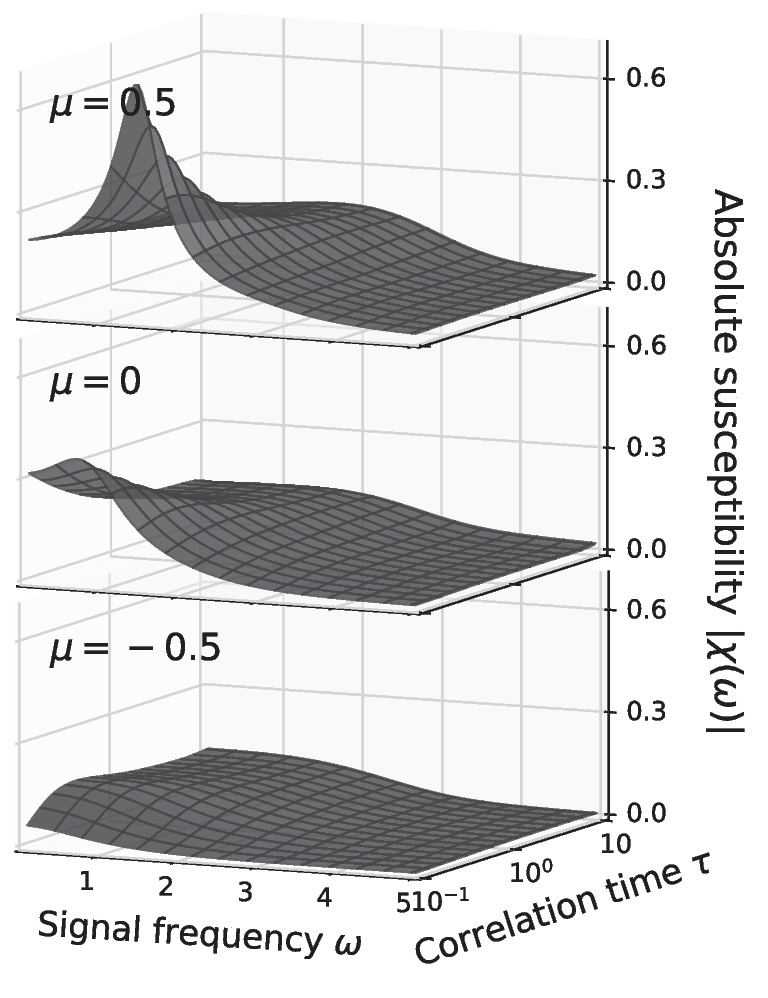
**Amplitude modulation** $\vert \chi (\omega )\vert$ **in the constant variance scaling** with $\sigma ^2 =1$ computed by the MCF method with $n_\text {max} = p_\text {max} =150$. For a discussion see the main text

**Fig. 11:**
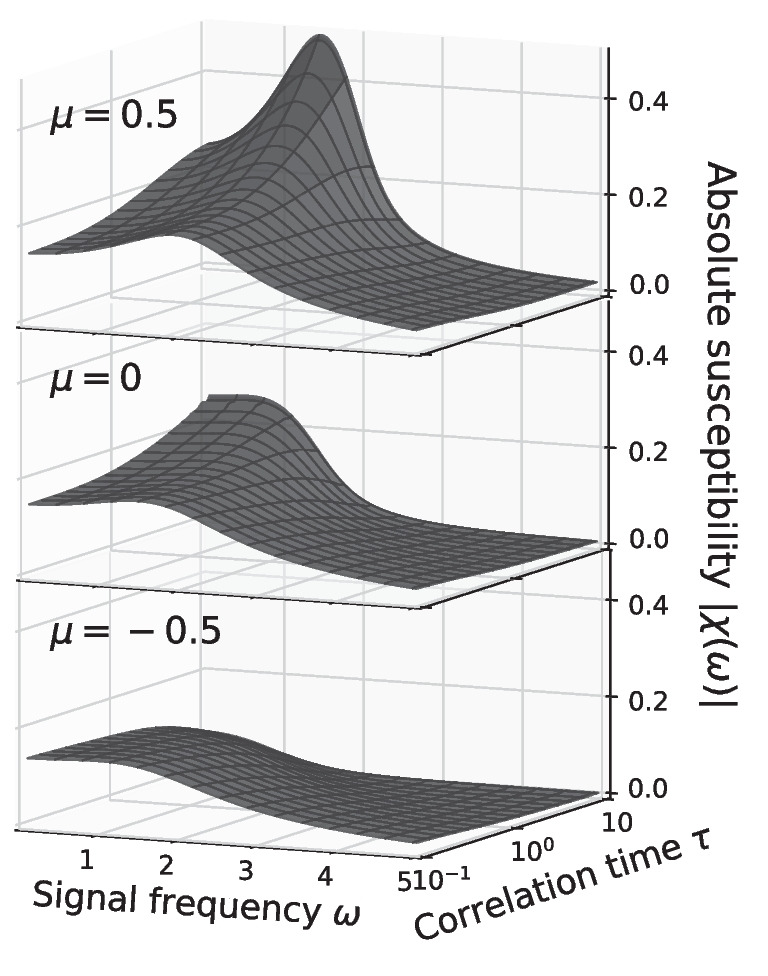
**Amplitude modulation** $\vert \chi (\omega )\vert$ **in the constant intensity scaling** with $D =1$ computed by the MCF method with $n_\text {max} = p_\text {max} = 150$. For a discussion see the main text

The general dependence of the susceptibility, focusing on its magnitude only, is inspected in Fig. 10 for the constant variance and in Fig. 11 for the constant intensity scaling. Qualitative different behavior of $\vert \chi (\omega )\vert$ can be observed between the mean-driven $\mu > 0$ and excitable regime $\mu < 0$. In the mean-driven regime the theta neuron exhibits a strong resonance near $\omega _\text {det} = 2 \pi r_\text {det}$ that increases with decreasing effect of the noise, i.e. in the constant variance scaling the resonance becomes stronger as $\tau \rightarrow 0$ (see Fig. 10 top) while for the constant intensity scaling the resonance increases as $\tau \rightarrow \infty$, (see Fig. 11 top).

In the excitable regime resonances are weak or absent. First of all, the baseline firing rate of the neuron vanishes as the effect of the noise decreases (cf. Figs. 3A and 7A) and so does the susceptibility (see Figs. 10 and 11 bottom). Secondly, the theta neuron becomes a low-pass filter where $\vert \chi (\omega )\vert$ decreases with increasing $\omega$ regardless of the correlation time $\tau$.

Right at the bifurcation point $\mu =0$ there are still no pronounced resonances with respect to $\omega$. However, the dependence of the linear response on the correlation time is somewhat different to the excitable regime: the susceptibility increases if the effect of the noise becomes very weak, i.e. $\tau \rightarrow 0$ for the constant variance scaling (see Fig. 10 middle) and $\tau \rightarrow \infty$ for the constant intensity scaling (see Fig. 11 middle).

### Nonlinear response

For larger signal amplitudes nonlinear response functions have to be considered:

52 $$\begin{aligned} r(t) = r_{0} &+ \varepsilon \vert r_{1,1}\vert \cos \left( \omega t - \varphi _{1,1}\right) \\& + \varepsilon ^2 \big [ r_{2,0} + \vert r_{2,2}\vert \cos \left( 2 \omega t - \varphi _{2,2 }\right) \big ] \\& + \varepsilon ^3 \big [\vert r_{3,1}\vert \cos \left( \omega t - \varphi _{3,1}\right) ) \\& \quad \quad + \vert r_{3,3}\vert \cos (3 \omega t - \varphi _{3,3})\big ] + ... \end{aligned}$$

Here we have included all terms up to the 3rd order in $\varepsilon$ (cf. Eq. (46)). The nonlinear response features higher Fourier modes and a correction $r_{2,0}$ of the time-averaged firing rate. The response functions $r_{\ell , k}$ and their respective argument $\varphi _{\ell ,k}$ of course depend on the model parameters $\mu$, $\tau$ and $\sigma$ as well as the signal frequency $\omega$.

**Fig. 12:**
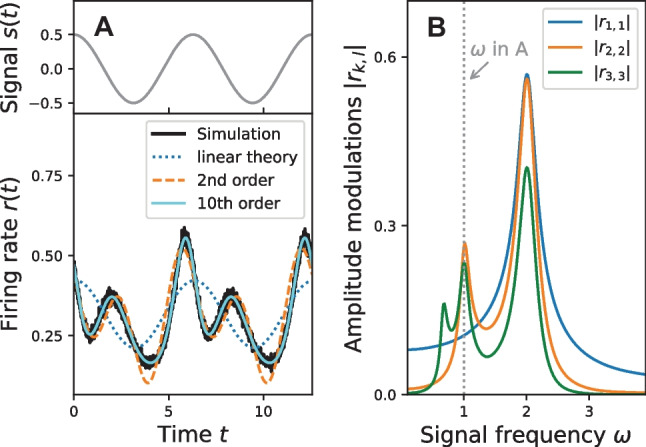
**Nonlinear response.** **A** Periodic signal and firing rate response of the theta neuron model. Here, the linear theory (dotted line) fails to accurately describe the firing rate (solid black line). This is mainly because the signal frequency is chosen to match half the deterministic firing frequency $\omega _\text {det} / 2 =1$ where the nonlinear response functions $\vert r_{2,2}\vert$ and $\vert r_{3,3}\vert$ are close to their local maximum, see **B**. However, already the second-order response (dashed orange line) provides a good approximation to the actual firing rate and is improved further if higher-order terms are considered (cyan line). All responses are calculated by the MCF method with $n_\text {max} = p_\text {max} =150$. Parameters: $\sigma = 1$, $\mu = 1$, $\tau = 0.1$, $\varepsilon = 0.5$ and $\omega =1$

For a neuron in the mean-driven regime the frequency dependence for three selected response functions is shown in Fig. 12B. In contrast to the linear response $\vert r_{1,1}\vert$ the functions $\vert r_{2,2}\vert$ and $\vert r_{3,3}\vert$ display additional resonances for instance at $\omega \approx 1 = \pi r_\mathrm{det}$. This behavior is not specific to the theta neuron, for instance such resonances can be observed for the LIF neuron as well (Voronenko & Lindner, 2017). These additional resonances give rise to strong nonlinear effects even if the signal is weak, see Fig. 12A. In the particular case shown in Fig. 12 the signal frequency was chosen to match the resonance frequency of the second-order response $\vert r_{2,2}\vert$ so that the linear response alone no longer provides a good approximation to the firing rate *r*(*t*). Instead the second-order response must be included, illustrating the importance of the nonlinear theory even for comparatively weak signals.

By means of the MCF method it is possible to achieve a near perfect fit of the actual firing rate by including many correction terms; see Fig. 12A where we have included all terms up to the 10th order. However, note that the computational cost of each further correcting term increases roughly linearly with the order $\ell$ of the signal amplitude.

**Fig. 13:**
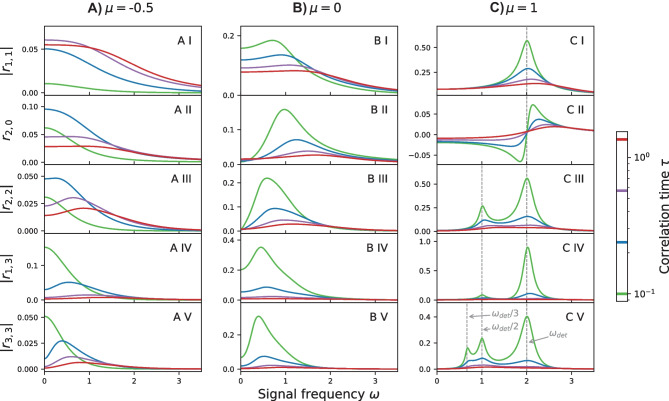
**Firing rate response functions** in the excitable regime (**A**), at the bifurcation point (**B**) and in the mean-driven regime (**C**) for a fixed variance $\sigma ^2 = 1$ and various values of $\tau$. For a discussion see the main text. Parameters MCF Method: $p_\text {max} = n_\text {max} = 200$

We now discuss the amplitude response functions $\vert r_{\ell ,k}\vert$ to the third order in $\varepsilon$ for varying values of the mean input and correlation time (cf. Fig. 13). The linear response $\vert r_{1,1}\vert$, already discussed in the preceding section and shown here for completeness (Fig. 13A I, B I, C I), displays in the mean-driven regime ($\mu =1$), and to a lesser degree also at the bifurcation point ($\mu =0$), a well known resonance peak near the firing frequency $\omega _0 = 2 \pi r_{0}$; it acts as a low-pass filter in the excitable regime ($\mu = -0.5$). Increasing the correlation time and thereby the effect of the noise diminishes this resonance.

The first nonlinear term $r_{2,0}$ describes the effect of the periodic signal on the time-averaged firing rate; we discuss this term first for the mean-driven regime (Fig. 13C II). Similar to the findings for a stochastic LIF model (Voronenko & Lindner, 2017, Fig. 3B) at low noise we find that a resonant driving at a frequency corresponding to the firing rate $\omega _0$ does not evoke any change of the time-averaged firing rate while a frequency slightly below or above this frequency evokes a reduction or increase of the rate, respectively. If we deviate too strongly from $\omega _0$ however the effect of the signal on the time-averaged rate becomes very small. Increasing the correlation time increases the effect of the noise and smears out these nonlinear resonances.

The effect of the periodic signal on the time-averaged firing rate in the excitable regime and at the bifurcation point is quite different (Fig. 13A II, B II). Here the rate is always increased by the periodic signal, similar to what was found already for an excitable LIF model (Voronenko & Lindner, 2017, Fig. 3A). Furthermore, at the bifurcation point and at low noise intensities (green curve in B II) there is a pronounced maximum as a function of frequency $\omega$ attained at a frequency higher than $\omega _0$.

Generally, in the higher-order response functions, we observe a number of peaks versus frequency (see e.g. Fig. 13A–C V). The resonances in the mean-driven regime (C V) and at low noise (green curve) are found near $\omega _\text {det}$, $\omega _\text {det}/2$ and $\omega _\text {det}/3$. Note again, that in this regime the deterministic frequency $\omega _\text {det}=2\pi r_\text {det}$ of the oscillator and the stationary firing frequency $\omega _0$ are close. In the excitable regime both the linear and nonlinear response functions also exhibit for most driving frequencies a nonmonotonic behavior with respect to the correlation time, i.e. with respect to the strength of the noise.

### Response to two periodic signals

So far we have discussed the theta neuron’s linear and nonlinear firing rate response to a *single* periodic signal. In this section we derive a scheme that allows to calculate the response if the model neuron receives two periodic signals:

53 $$\begin{aligned} s(t) &{}= s_1(t) + s_2(t) \\ &{}=\varepsilon _1 \cos (\omega _1 t) + \varepsilon _2 \cos (\omega _2 t). \end{aligned}$$

Calculating the firing rate in this case will not only help to understand how a theta neuron responds to two periodic signals but can also be used to calculate the 2nd order response to arbitrary signals (Voronenko & Lindner, 2017).

As a starting point we formulate the corresponding FPE:

54 $$\begin{aligned} \partial _t P(\theta ,\eta ,t) = \left( \hat{L}_0(\theta ,\eta ) - s(t) \hat{L}_\text {per}(\theta ) \right) P(\theta ,\eta ,t). \end{aligned}$$

This equation still agrees with Eq. (41) except for *s*(*t*) which contains two periodic signals. Again we are interested in the PDF for which all initial condition have been forgotten and the time dependence of $P(\theta ,\eta ,t)$ is only due to the time dependence of the signal *s*(*t*). Note that since the sum of two periodic signals is not necessarily periodic, the functions $P(\theta ,\eta ,t)$ and *r*(*t*) are not periodic either. In fact, *s*(*t*) is only periodic if the ratio of the two frequencies is a rational number, i.e. $\omega _1/\omega _2 \in \mathbb {Q}$. We chose a Fourier representation with respect to $\omega _1 t$, $\omega _2 t$ and expand with respect to the small amplitudes $\varepsilon _1$, $\varepsilon _2$:

55 $$\begin{aligned} P(\theta ,\eta ,t) = \sum _{\begin{array}{c} \ell _1 = 0 \\ \ell _2 = 0 \end{array}}^\infty \sum _{\begin{array}{c} k_1 = -\infty \\ k_2 = -\infty \end{array}}^\infty \varepsilon _1^{\ell _1} \varepsilon _2^{\ell _2} e^{-i (k_1 \omega _1 + k_2 \omega _2) t} P_{k_1,k_2}^{\ell _1,\ell _2}. \end{aligned}$$

For notational convenience we have omitted the arguments of the coefficients $P_{k_1,k_2}^{\ell _1,\ell _2}(\theta , \eta )$.

Because $P(\theta ,\eta ,t)$ is a real valued function, the coefficients obey

56 $$\begin{aligned} P_{k_1,k_2}^{\ell _1,\ell _2} = \left( P_{-k_1,-k_2}^{\ell _1,\ell _2} \right) ^*. \end{aligned}$$

As for the case of a single periodic signal, inserting Eq. (55) into Eq. (54) gives a system of time-independent coupled differential equations:

57 $$\begin{aligned} \begin{aligned} \hat{L}_{k_1,k_2} P_{k_1,k_2}^{\ell _1,\ell _2} = \frac{1}{2}\hat{L}_\text {per}&\left( P_{k_1+1,k_2}^{\ell _1-1,\ell _2} + P_{k_1-1,k_2}^{\ell _1-1,\ell _2} + \right. \\ {}&\left. + P_{k_1,k_2+1}^{\ell _1,\ell _2-1} + P_{k_1,k_2-1}^{\ell _1,\ell _2-1}\right) \end{aligned} \end{aligned}$$

with $\hat{L}_{k_1,k_2} = \hat{L}_0 + i(k_1\omega _1 + k_2 \omega _2)$ and $P_{k_1,k_2}^{\ell _1,\ell _2}= 0$ for $\ell _1 < 0$ or $\ell _2 < 0$. The normalization of the probability density provides again the additional conditions:

58 $$\begin{aligned} \int _{-\pi }^{\pi } d\theta \int _{0}^{\infty } d\eta \,P_{k_1,k_2}^{\ell _1,\ell _2} = \delta _{k_1, 0} \delta _{k_2, 0} \delta _{\ell _1, 0} \delta _{\ell _2, 0}. \end{aligned}$$

The differential equations (57) are analogous to Eq. (44) and can be solved by means of the MCF method (see Appendix C). In the following we explicitly provide the hierarchy of coupled differential equations up to the second order of $\varepsilon _1, \varepsilon _2$, i.e. for $\ell _1 + \ell _2 \le 2$. The zeroth-order term $\ell _1 + \ell _2 = 0$ describes the unperturbed system. As we have already argued for the case of a single periodic signal the function $P_{0,0}^{0,0}$, governed by

59 $$\begin{aligned} \hat{L}_{0,0} P_{0,0}^{0,0} = 0, \end{aligned}$$

is the only non-vanshing zeroth-order term because for every other value of $k_1, k_2$ the trivial solution does satisfies Eq. (58). Therefore $P_{0,0}^{0,0} = P_0$ is the stationary probability density from Sect. 3. The stationary PDF in turn determines the two non-vanishing linear ($\ell _1 + \ell _2 = 1$) correction terms:

60 $$\begin{aligned} \hat{L}_{1,0} P_{1,0}^{1,0}&= \frac{\hat{L}_\text {per}}{2} P_{0,0}^{0,0},\end{aligned}$$

61 $$\begin{aligned} \hat{L}_{0,1} P_{0,1}^{0,1}&= \frac{\hat{L}_\text {per}}{2} P_{0,0}^{0,0}. \end{aligned}$$

Finally, the linear terms determine the second order terms ($\ell _1 + \ell _2 = 2$):

62 $$\begin{aligned} \hat{L}_{2,0} P_{2,0}^{2,0}&= \frac{\hat{L}_\text {per}}{2} P_{1,0}^{1,0}, \end{aligned}$$

63 $$\begin{aligned} \hat{L}_{0,2} P_{0,2}^{0,2}&= \frac{\hat{L}_\text {per}}{2} P_{0,1}^{0,1}, \end{aligned}$$

64 $$\begin{aligned} \hat{L}_{0,0} P_{0,0}^{0,2}&= \frac{\hat{L}_\text {per}}{2} \left[ P_{0,1}^{0,1} + (P_{0,1}^{0,1})^* \right] , \end{aligned}$$

65 $$\begin{aligned} \hat{L}_{0,0} P_{0,0}^{2,0}&= \frac{\hat{L}_\text {per}}{2} \left[ P_{1,0}^{1,0} + (P_{1,0}^{1,0})^* \right] , \end{aligned}$$

66 $$\begin{aligned} \hat{L}_{1,-1} P_{1,-1}^{1,1}&= \frac{\hat{L}_\text {per}}{2} \left[ P_{1,0}^{1,0} + (P_{0,1}^{0,1})^* \right] ,\end{aligned}$$

67 $$\begin{aligned} \hat{L}_{1,1} P_{1,1}^{1,1}&= \frac{\hat{L}_\text {per}}{2} \left[ P_{1,0}^{1,0} + P_{0,1}^{0,1} \right] . \end{aligned}$$

**Fig. 14:**
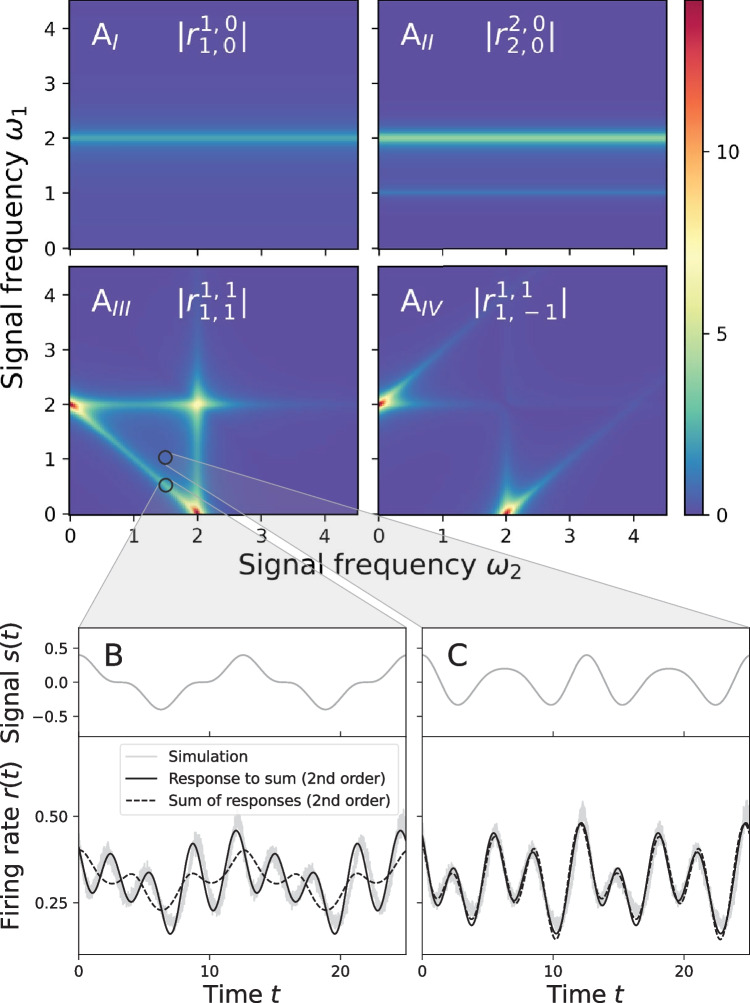
Nonlinear response to two periodic signals. A$_I$-A$_{IV}$) Amplitudes of the response functions $r_{l_1,l_2}^{k_1,k_2}$ (cf. Eq. (71)). Note that the response functions $\vert r_{0,1}^{0,1}\vert$ and $\vert r_{0,2}^{0,2}\vert$ that are not shown here are identical to the response functions that are shown in A$_I$ and A$_{II}$ if the frequencies $\omega _1$ and $\omega _2$ are interchanged (both account for a single signal). The response functions $\vert r_{1,1}^{1,1}\vert$ and $\vert r_{1,-1}^{1,1}\vert$, that describe the interaction effect of both signals on the firing rate, exhibit additional resonances near $\omega _1 + \omega _2 = 2 \pi r_\text {det}$ and $\vert \omega _1 - \omega _2\vert = 2 \pi r_\text {det}$. B and C show the firing rate in response to two periodic signals where the sum of the frequencies does and does not match the aforementioned condition $\omega _1 + \omega _2 = 2 \pi r_\text {det}$, respectively. If the condition is matched the sum of responses to each individual signal does not provide a good approximation to the actual firing rate but the full response to the sum of signals has to be calculated (see B). Parameters: $\mu = 1$, $\sigma ^2 = 1$, $\tau =0.05$, $\varepsilon _1 = 0.3$, $\varepsilon _2 = 0.1$ with frequencies $\omega _1 =0.5$, $\omega _2 = 1.5$ in B and $\omega _1 =1.0$, $\omega _2 = 1.5$ in C. Parameters MCF Method: $p_\text {max} = n_\text {max} = 100$

As for the case of a single periodic signal, the rate response *r*(*t*) can be expressed in terms of the functions $P_{k_1,k_2}^{\ell _1,\ell _2}$ using Eqs. (21) and (56):

68 $$\begin{aligned} r(t) = \sum _{\ell _1,\ell _2} &{}\sum _{k_1,k_2} \varepsilon _1^{\ell _1} \varepsilon _2^{\ell _2} \vert r_{k_1,k_2}^{\ell _1,\ell _2}\vert \times \\ &{}\times \cos \left( (k_1 \omega _1 + k_2 \omega _2) t - \varphi _{k_1,k_2}^{\ell _1,\ell _2} \right) , \end{aligned}$$

with

69 $$\begin{aligned} r_{k_1,k_2}^{\ell _1,\ell _2}&= 2(2 - \delta _{k_1,0}\delta _{k_2,0}) \int \limits _{-\infty }^\infty d\eta \, P_{k_1,k_2}^{\ell _1,\ell _2}(\pi ,\eta ), \end{aligned}$$

70 $$\begin{aligned} \varphi _{k_1,k_2}^{\ell _1,\ell _2}&= \arg (r_{k_1,k_2}^{\ell _1,\ell _2}). \end{aligned}$$

The response of the firing rate up to the second order in the amplitudes reads:

71 $$\begin{aligned} r(t) \approx \;& r_{0,0}^{0,0}\\ &{}+ \varepsilon _1 \vert r_{1,0}^{1,0}\vert \cos \left( \omega _1 t - \varphi _{1,0}^{1,0}\right) \\ &{}+ \varepsilon _2 \vert r_{0,1}^{0,1}\vert \cos \left( \omega _2 t - \varphi _{0,1}^{0,1}\right) \\ &{}+ \varepsilon _1^2 \left[ r_{0,0}^{2,0} + \vert r_{2,0}^{2,0}\vert \cos \left( 2\omega _1 t - \varphi _{2,0}^{2,0}\right) \right] \\ &{}+ \varepsilon _2^2 \left[ r_{0,0}^{0,2} + \vert r_{0,2}^{0,2}\vert \cos \left( 2\omega _2 t - \varphi _{0,2}^{0,2}\right) \right] \\ &{}+ \varepsilon _1 \varepsilon _2 \left[ \vert r_{1,1}^{1,1}\vert \cos \left( (\omega _1+\omega _2) t - \varphi _{1,1}^{1,1}\right) \right. \\ &{}\qquad + \left. \vert r_{1,-1}^{1,1}\vert \cos \left( (\omega _1-\omega _2) t - \varphi _{1,-1}^{1,1}\right) \right] . \end{aligned}$$

The first five lines represent the first and second order responses of the firing rate for a theta neuron that receives a single periodic signal, either $s_1(t)$ or $s_2(t)$. For instance, $\vert r_{1,0}^{1,0}(\omega _1,\omega _2)\vert = \vert r_{1,1}(\omega _1)\vert$ (the linear response amplitude to $s_1$) and $\vert r_{2,0}^{2,0}(\omega _1,\omega _2)\vert = \vert r_{2,2}(\omega _1)\vert$ (the response amplitude at the second harmonic of $s_1$) do not depend on the frequency of the second signal $\omega _2$ as it can be seen in Fig. 14A$_I$ and A$_{II}$. The response functions $r_{\ell ,k}(\omega )$ for a single periodic signal have already been discussed in the previous sections. The last two terms, proportional to $\varepsilon _1 \varepsilon _2$, are of particular interest here, because they arise only due to the interaction of *two* periodic signals. The corresponding response amplitudes $\vert r_{1,1}^{1,1}\vert$ and $\vert r_{1,1}^{1,-1}\vert$ are shown in Fig. 14A$_{III}$ and A$_{IV}$. In accordance with previous observations for the leaky integrate-and-fire model with white noise and a periodic driving (Voronenko & Lindner, 2017) we find two distinct cases in the mean-driven regime. First, if neither the sum nor the difference $\omega _1 \pm \omega _2$ is close to the firing frequency $2 \pi r_\text {det}$ then the response to the sum of two signals is well described by the sum of responses to the separate signals. A particular set of frequencies $\omega _1$ and $\omega _2$ for which this is the case is shown in Fig. 14C where the second order response to the sum of two signals (black solid line) agrees very well with the sum of the second order responses to one signal at a time (dashed line). Second, if $\omega _1 + \omega _2 \approx 2 \pi r_\text {det}$ or $\vert \omega _1 - \omega _2\vert \approx 2 \pi r_\text {det}$ the firing rate is significantly affected by the interaction of both signals (see Fig. 14A$_{III}$ and A$_{IV}$). An example of the firing rate as a function of time where these interaction terms are crucial is shown in Fig. 14B. Here the aforementioned response to the sum of two signals and sum of responses to one signal at a time disagree significantly.

## Summary and outlook

In this paper we have studied the firing rate of the canonical type-I neuron model, the theta neuron, subject to a temporally correlated Ornstein-Uhlenbeck noise and additional periodic signals. We have solved the associated multi-dimensional Fokker-Planck-equation numerically by means of the matrix-continued-fraction (MCF) method, put forward by Risken (1984). For our problem the MCF method provided reliable solutions for a wide range of parameters; the main restriction is that the correlation time cannot be to large and additionally in the excitable regime the noise intensity (as also known from other application of the method, see Lindner & Sokolov, 2016 for a recent example) cannot be to small. To the best of our knowledge this is the first application of this method in computational neuroscience, advancing the results by Naundorf et al. (2005a, b) on the same model.

When the neuron receives no additional periodic signal, i.e. when the model is driven solely by the correlated noise, our method allows a quick and accurate computation of the stationary firing rate. We investigated the rate for a large part of the parameter space, confirmed the MCF results by comparison with stochastic simulations and discussed the agreement with known analytical approximations (Fourcaud-Trocmé et al., 2003; Galán, 2009; Moreno-Bote & Parga, 2010). We found that, in contrast to the white noise case (Lindner et al., 2003), correlated noise can both increase and decrease the stationary firing rate of a type-I neuron and we identified the conditions under which one or the other behavior can be observed.

In the presence of a single additional periodic signal both the probability density function and the firing rate approach a cyclo-stationary solution, which can be found by extending the MCF method to the time-dependent Fokker-Planck-equation. The corresponding rate modulation is for a weak signal given by the linear response function, the well known susceptibility, which has been addressed before numerically (Naundorf et al., 2005a, b) and analytically in limit cases (Fourcaud-Trocmé et al., 2003). Here we went beyond the linear response and computed also the higher-order response to a single periodic stimulus. Similar to what was found for a periodically driven leaky integrate-and-fire model with white background noise (Voronenko & Lindner, 2017), we identified driving frequencies at which the higher harmonics can be stronger than the firing rate modulation with the fundamental frequency. For a variety of nonlinear response functions, we observed resonant behavior.

Finally, we generalized the numerical approach to the case of two periodic signals and studied the nonlinear response up to second order. We found that for certain frequency combinations the mixed response to the two signals can lead to a drastically different rate modulation than predicted by pure linear response theory; this is similar to what was observed in a leaky integrate-and-fire neuron with white background noise (Voronenko & Lindner, 2017).

Our method could be extended to neuron models that include more complicated correlated noise, for instance, a harmonic noise (Schimansky-Geier & Zülicke, 1990) that can mimic special sources of intrinsic fluctuations (Engel et al., 2009). Another problem that could be addressed by this method is the computation of the spike-train power spectrum in the stationary state. Furthermore the linear and nonlinear response to the modulation of other parameters, e.g. the noise intensity (Boucsein et al., 2009; Lindner & Schimansky-Geier, 2001; Silberberg et al., 2004; Tchumatchenko et al., 2011), could be of interest and be computed with the methods outlined in this paper.

## Figures and Tables

**Fig. 15:**
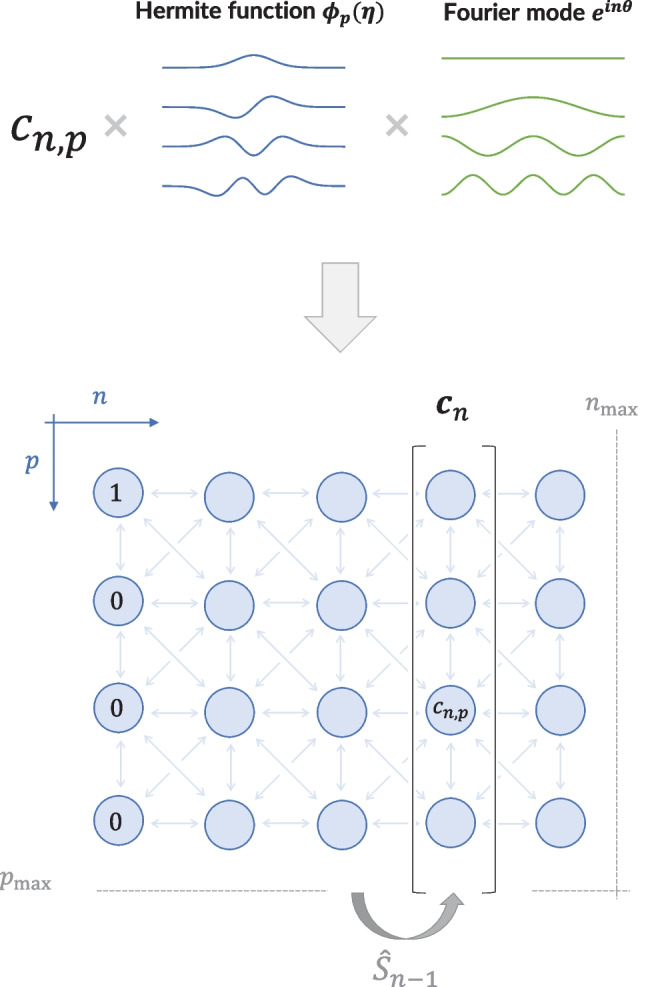
**Illustration of the MCF method.** Expanding the probability density using eigenfunctions according to Eq. (75) and insertion into the FPE (72) leads to a relation for the expansion coefficients $c_{n,p}$ where only nearest neighbors interact (including diagonals). The coefficients can then be computed by truncating the system and introducing transition matrices $\hat{S}_n$ that can be obtained from a matrix continued fraction, see Sect. 3.1

**Fig. 16:**
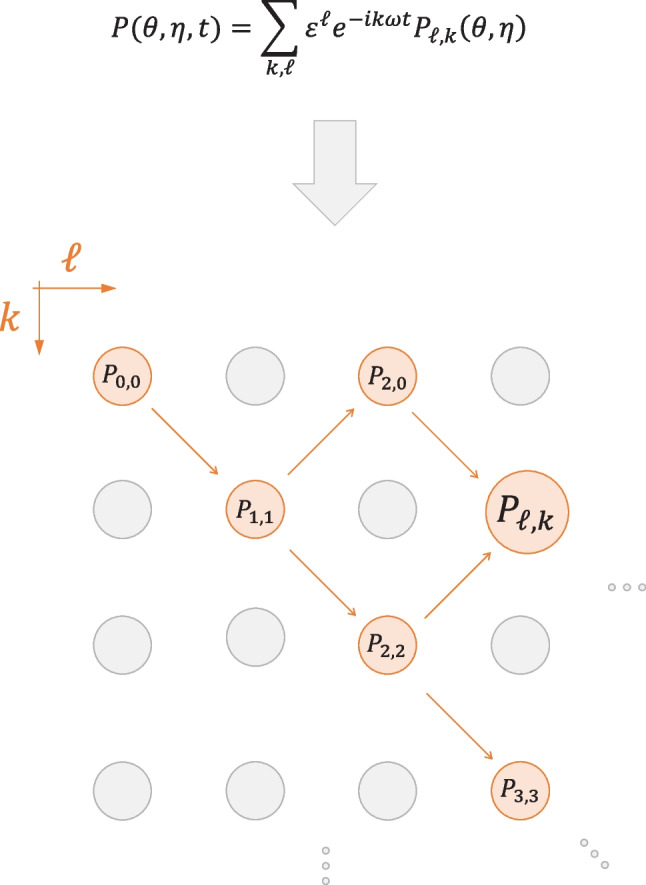
**Expansion of the PDF and coupling hierarchy.** If the theta neuron is subject to a periodic signal the cyclo-stationary PDF can be expanded according to Eq. (94). Inserting this ansatz into the FPE leads to a system of time-independent recursively coupled differential equations (93). The coupling hierarchy is shown here. The stationary PDF $P_{0,0}$ determines $P_{1,1}$ which in turn determines $P_{2,0}$ and $P_{2,2}$ and so on. Gray dots represent terms that vanish as explained in Sect. 4

## A. Stationary case - derivation of the tridiagonal recurrence relation

Here we demonstrate how the problem of solving the FPE (22) for the stationary PDF $P_0(\theta , \eta )$, can be translated into an equivalent problem of solving a tridiagonal recurrence relation for the expansion coefficients $c_p^n$. These coefficients can then be found by means of the matrix-continued-fraction method as it was demonstrated in Sect. 3.1.

First, we recall the stationary FPE

72 $$\begin{aligned} 0&= \hat{L}_0 P_0(\theta ,\eta ) =(\hat{L}_\theta + \hat{L}_\eta ) P_0(\theta ,\eta ), \end{aligned}$$

73 $$\begin{aligned} \hat{L}_\theta&= - \partial _\theta f_0(\theta ,\eta ) \end{aligned}$$

74 $$\begin{aligned} \hat{L}_\eta&= \frac{1}{\tau } \partial _\eta (\eta + \sigma ^2 \partial _\eta ), \end{aligned}$$

and the expansion of the PDF by two sets of orthonormal eigenfunctions $e^{i n \theta }/ \sqrt{2 \pi }$ and $\phi _p(\eta )$

75 $$\begin{aligned} P_0(\theta ,\eta ) = \frac{\phi _0(\eta )}{2 \pi } \sum _{p=0}^{\infty } \sum _{n=-\infty }^{\infty } c_{n,p} e^{i n \theta } \phi _p(\eta ). \end{aligned}$$

The Fourier modes and Hermite functions satisfy the periodic boundary condition in $\theta$ and natural boundary conditions in $\eta$, respectively. Using the orthonormality

76 $$\begin{aligned} \frac{1}{2 \pi } \int \limits _{-\pi }^{\pi } d\theta \, e^{i n \theta } e^{i m \theta }&= \delta _{n,m},&\int \limits _{-\infty }^{\infty } d\eta \, \phi _p \phi _q&= \delta _{p,q} \end{aligned}$$

of these functions one can show that the normalization condition of the PDF determines $c_{0,0}$:

77 $$\begin{aligned} c_{0,0} = \int \limits _{-\pi }^{\pi } d\theta \int \limits _{-\infty } ^{\infty } d\eta P(\theta ,\eta ,t) = 1 \end{aligned}.$$

Before addressing the full problem of finding the recursive relation for the coefficients $c_{n,p}$, we first calculate $\hat{L}_\eta P_0(\theta ,\eta )$ using the expansion (75):

78 $$\begin{aligned} \hat{L}_\eta P_0 = \frac{1}{2\pi }\sum _{n,p} c_{n,p} e^{in\theta } \hat{L}_\eta \phi _0(\eta ) \phi _p(\eta ) \end{aligned}.$$

Remember that the Hermite functions can expressed by the Hermite polynomials $H_p(x)$ as follows:

79 $$\begin{aligned} \phi _p(\eta ) = \frac{H_p(\eta /\alpha )}{\sqrt{2^p p! \sqrt{\pi } \alpha }} e^{-\frac{\eta ^2}{2\alpha ^2}}. \end{aligned}$$

Where $\alpha$ is an arbitrary scaling factor. Making use of the two properties

80 $$\begin{aligned} \eta \phi _p&= \frac{\alpha }{\sqrt{2}} \left( \sqrt{p} \phi _{p-1}+ \sqrt{p+1} \phi _{p+1} \right) , \end{aligned}$$

81 $$\begin{aligned} (\phi _0 \phi _p)'&= - \frac{\sqrt{2}}{\alpha } \sqrt{p+1} \phi _0 \phi _{p+1} \end{aligned}$$

of the Hermite functions we can derive a handy expression for $\hat{L}_\eta \phi _0(\eta ) \phi _p(\eta )$ by choosing $\alpha = \sqrt{2}\sigma$:

82 $$\begin{aligned} \hat{L}_\eta \phi _0 \phi _p&= \frac{\partial _\eta }{\tau } \left[ \eta + \sigma ^2 \partial _\eta \right] \phi _0 \phi _p \nonumber \\&= \frac{ \partial _\eta }{\tau } \frac{\alpha }{\sqrt{2}} \phi _0 \left[ \sqrt{p} \phi _{p-1}+\left( 1- \tfrac{4\sigma ^2}{a^2} \right) \sqrt{p+1} \phi _{p+1} \right] \nonumber \\&= - \frac{p}{\tau } \phi _0 \phi _p. \end{aligned}$$

Hence, $\phi _0 \phi _p$ is a eigenfunction of the operator $\hat{L}_\eta$. Combining Eq. (82), the expansion (75) and the FPE (72) yields

83 $$\begin{aligned} 0 = \sum _{n,p} c_{n,p} \, \phi _p \{ &{}[i p / (n \tau ) - 1 - \mu - \eta ] n e^{in\theta }\\ + \frac{1}{2}&{}[1-\mu -\eta ](n+1)e^{i(n+1)\theta } \\ + \frac{1}{2}&{}[1-\mu -\eta ] (n-1)e^{i(n-1)\theta } \}. \end{aligned}$$

We split the sum into three parts, perform an index shift and use Eq. (80) to obtain

84 $$\begin{aligned} 0 = &{}\sum _{n,p} n e^{in\theta } \{ [(i p / (n \tau ) - 1 - \mu ) \phi _p \\ &{}-\sigma (\sqrt{p+1}\phi _{p+1} + \sqrt{p}\phi _{p-1}) ] c_{n,p} \\ &{} + \frac{1}{2}[(1-\mu ) \phi _p - \sigma ( \sqrt{p+1}\phi _{p+1} + \sqrt{p}\phi _{p-1})]c_{n+1,p} \\ &{} + \frac{1}{2}[(1-\mu ) \phi _p - \sigma ( \sqrt{p+1}\phi _{p+1} + \sqrt{p}\phi _{p-1})]c_{n-1,p} \}. \end{aligned}$$

Furthermore, we introduce the orthonormal operators

85 $$\begin{aligned} \hat{O}_\theta&= \frac{1}{2\pi }\int _{0}^{2 \pi } d\theta \, e^{i n \theta },&\hat{O}_\eta&= \int _{-\infty }^{\infty } d\eta \phi _q(\eta ) \end{aligned}$$

so that $\hat{O}_\theta e^{im\theta } = \delta _{n,m}$ and $\hat{O}_\eta \phi _p = \delta _{p,q}$. Multiplying Eq. (84) from the left by $\hat{O}_\eta \hat{O}_\theta$ allows to get rid of the sum over *n* and to find the following recursive relation.

86 $$\begin{aligned} 0=&{}n \sum _{p} \{ [ (i p / (n \tau ) - 1 - \mu )\delta _{p,q} \\ &{}-\sigma (\sqrt{q} \delta _{p+1,q} + \sqrt{q+1}\delta _{p-1,q})] c_{n,p} \\ &{} + \frac{1}{2}[(1-\mu ) \delta _{p,q} \sigma ( \sqrt{q} \delta _{p+1,q} + \sqrt{q+1}\delta _{p-1,q})] c_{n+1,p} \\ &{} + \frac{1}{2}[(1-\mu ) \delta _{p,q} \sigma ( \sqrt{q} \delta _{p+1,q} + \sqrt{q+1}\delta _{p-1,q})] c_{n-1,p} \} \end{aligned}$$

The sum can be interpreted as a product of matrices and vectors. We introduce the coefficient vector

87 $$\begin{aligned} \varvec{c}_n = \left( \begin{array}{c} c_{n,0}\\ c_{n,1}\\ ... \end{array} \right) \end{aligned}$$

and the symmetric matrices

88 $$\begin{aligned} \quad \left( \hat{A} \right) _{p,q}&= i \frac{q}{\tau } \delta _{p,q}, \end{aligned}$$

89 $$\begin{aligned} \quad \left( \hat{B} \right) _{p,q}&= \frac{1-\mu }{2} \delta _{p,q} - \frac{\sigma }{2} \left( \sqrt{q} \delta _{p+1,q} + \sqrt{q+1} \delta _{p-1,q} \right) , \end{aligned}$$

which allows for an elegant reformulation of Eq. (86) by the tridiagonal recurrence relation

90 $$\begin{aligned} 0 = \left( \hat{A} + 2 n(\hat{B}- \mathbbm {1}) \right) \boldsymbol{c}_n + n \hat{B} \left( \boldsymbol{c}_{n-1} + \boldsymbol{c}_{n+1} \right) . \end{aligned}$$

Note, that for $n=0$ we can readily infer the remaining elements of $c_0$ (remember that $c_{0,0} = 1$)

91 $$\begin{aligned} \varvec{c}_0 = \left( 1,0,0, ... \right) ^T. \end{aligned}$$

Equation (90) can by simplified by multiplication with $\hat{B}^{-1}/n$ from the left to obtain the expression used in Sect. 3.1:

92 $$\begin{aligned} \hat{K}_n \varvec{c}_n = \varvec{c}_{n-1} + \varvec{c}_{n+1}. \end{aligned}$$

This is the tridiagonal recurrence relation which we have solved in the main part by the MCF method (illustrated in Fig. 15).

## B. Cyclo-stationary case - MCF method

In this section we expand the MCF method to the case of an additional periodic signal $s(t) = \varepsilon \cos (\omega t)$ and calculate the cyclo-stationary firing rate *r*(*t*). In Sect. 4 we have already shown that the time-dependent FPE for this problem, i.e. Eq. (41), can be transformed into a set of time-independent differential equations that are recursively related (cf. Eq. (44)):

93 $$\begin{aligned} \left( \hat{L}_0 + ik\omega \right) P_{\ell , k} = \tfrac{\hat{L}_\text {per}}{2}(P_{\ell -1, k-1} + P_{\ell -1, k+1}), \end{aligned}$$

with $P_{\ell <0, k} = 0$. This hierarchy of coupled differential equations can be solved iteratively starting at the stationary PDF $P_{0,0}$. The dependence is illustrated in Fig. 16 where many terms $P_{\ell ,k}$ vanish (grey circles) due to the normalization condition of the PDF as explained in Sect. 4. In order to solve the corresponding differential equation for each $P_{\ell , k}(\theta ,\eta )$ we chose the same ansatz as in the previous section:

94 $$\begin{aligned} P_{\ell ,k}(\theta ,\eta ) = \frac{\phi _0(\eta )}{2 \pi } \sum _{p=0}^{\infty } \sum _{n=-\infty }^{\infty } c_{n,p}^{(\ell ,k)} e^{i n \theta } \phi _p(\eta ). \end{aligned}$$

By substituting this ansatz into Eq. (47) a relation between the expansion coefficients $c_{n, p}^{(\ell ,k)}$ and response functions $r_{\ell ,k}$ (which determine the full firing rate *r*(*t*) via Eq. (46)) can be derived:

95 $$\begin{aligned} r_{\ell ,k} = \frac{(2 - \delta _{k,0})}{\pi } \sum _{n = -\infty }^{\infty } (-1)^n c_{n,0}^{(\ell , k)}. \end{aligned}$$

To find $c_{n, p}^{(\ell ,k)}$, we again transform the differential equations (93) into coefficient equations by means of the expansion (94) (see derivation of the tridiagonal recurrence relation in the previous section) and obtain

96 $$\begin{aligned} 0 =& \left( \hat{A} + k \omega \mathbbm {1} + 2 n(\hat{B}- \mathbbm {1}) \right) \boldsymbol{c}_{n} \\ &{}+ n \hat{B} \left( \boldsymbol{c}_{n-1}+ \boldsymbol{c}_{n+1} \right) \\ &{}- \frac{n}{4}\left( 2 \boldsymbol{c}_{n}' + \boldsymbol{c}_{n-1}' + \boldsymbol{c}_{n+1}' \right) . \end{aligned}$$

For notational convenience we introduced the coefficient vectors

97 $$\begin{aligned} \varvec{c}_n^{(\ell ,k)} = \left( c_{n,0}^{(\ell ,k)}, c_{n,1}^{(\ell ,k)}, ... \right) ^T, \end{aligned}$$

and droped the superscripts $\varvec{c}_n := \varvec{c}_n^{(\ell ,k)}$. Further we denote the sum of the *previously* computed coefficient vectors by $\varvec{c}_n' := \varvec{c}_n^{(\ell -1,k+1)} + \varvec{c}_n^{(\ell -1,k-1)}$.

As in the previous section Eq. (96) is multiplied by $\hat{B}^{-1}/n$ from the left to obtain a more compact expression

98 $$\begin{aligned} \hat{K}_n \varvec{c}_n = \varvec{c}_{n+1} +\varvec{c}_{n-1} + \varvec{\tilde{c}}_{n} \end{aligned}$$

with

99 $$\begin{aligned} \hat{K}_n&= 2 \left( \hat{B}^{-1}-\mathbbm {1} \right) - \frac{\hat{B}^{-1} (\hat{A}+k\omega \mathbbm {1})}{n} \end{aligned}$$

100 $$\begin{aligned} \varvec{\tilde{c}}_{n}&= -\frac{\hat{B}^{-1}}{4} ( 2\varvec{c}_n' + \varvec{c}_{n+1}' + \varvec{c}_{n-1}' ). \end{aligned}$$

In order to solve the 2-dimensional coefficient equation (98) we must assume that all Hermite functions and Fourier modes become negligible for large *p* or *n*, so that the corresponding coefficients vanish: $c_{n,p} := c_{n,p}^{(\ell ,k)} = 0$ for $p>p_\mathrm{max}.$ or $n>n_\mathrm{max}$. Specifically, we have checked how key statistics as for instance the firing rate depend on *p* and *n* and observed saturation for sufficiently large *p* and *n*; we then take these as maximal values.

The normalization condition of the PDF is determines the coefficient $c_{0,0} := c_{0,0}^{(\ell ,k)}$:

101 $$\begin{aligned} \int _{-\pi }^{\pi } d\theta \int _{-\infty } ^{\infty } d\eta P_{\ell , k}(\theta ,\eta ) = c_{0,0} = \delta _{k,0}\delta _{\ell , 0} \end{aligned}$$

The remaining elements of $\varvec{c}_{0}$ vanish. This can be seen from Eq. (96) that simplifies considerably for $n = 0$

102 $$\begin{aligned} \left( \hat{A} + k \omega \mathbbm {1} \right) \boldsymbol{c}_{0} = 0. \end{aligned}$$

The involved matrix $A_k = \hat{A} + k \omega \mathbbm {1}$ is diagonal with non-vanishing elements $(A_k)_{p,p} \ne 0$ for $p \ne 0$. This implies that Eq. (102) can only be fulfilled if $c_{p\ne 0,0} = 0$. The resulting coefficient vector

103 $$\begin{aligned} \varvec{c}_{0} = {\left\{ \begin{array}{ll} (1, ...,0)^T &{}\text {if } k = \ell = 0\\ (0, ...,0)^T &{}\text {else}\\ \end{array}\right. } \end{aligned}$$

serves as the initial condition in the following. All other coefficient vectors can be derived iteratively:

104 $$\begin{aligned} \varvec{c}_{n+1} = \hat{S}_{n} \varvec{c}_{n} + \varvec{d}_n, \qquad \text {for } n = 0, ...,n_\text {max}-1 \end{aligned}$$

105 $$\begin{aligned} \varvec{c}_{n-1} = \hat{S}_{n}^R \varvec{c}_{n} + \varvec{d}^R_n, \qquad \text {for } n = 0, ..., -n_\text {max}+1, \end{aligned}$$

Here we have introduced the transition matrices $\hat{S}_n$ and $\hat{S}_n^R$ as done in the case of no periodic signal and the additional vectors $\varvec{d}_n$ and $\varvec{d}_n^R$, which take the inhomogeneity $\varvec{\tilde{c}}_n$ in Eq. (98) into account (Risken, 1984). The ansatz Eq. (104) is substituted into Eq. (98), which yields

106 $$\begin{array}{l} [( \hat{K}_n-\hat{S}_n ) \hat{S}_{n-1} - \mathbbm {1}] \boldsymbol{c}_{n-1} = \\ \qquad\qquad [\boldsymbol{d}_{n} - ( \hat{K}_n-\hat{S}_n ) \boldsymbol{d}_{n-1} + {\tilde{\boldsymbol{c}}}_n]. \end{array}$$

This equation is satisfied when both expressions in the square brackets vanish. This allows to derive two recursive relation. First, from the left hand side

107 $$\begin{aligned} \hat{S}_{n-1} = [\hat{K}_n -\hat{S}_n ]^{-1} \end{aligned}$$

and second, from the right hand side

108 $$\begin{aligned} \begin{aligned} \varvec{d}_{n-1}&= [\hat{K}_n -\hat{S}_n ]^{-1} (\varvec{d}_n + \varvec{\tilde{c}}_{n})\\ {}&= \hat{S}_{n-1} ( \varvec{d}_n + \varvec{\tilde{c}}_{n} ). \end{aligned} \end{aligned}$$

Analogous expressions can be derived for $\hat{S}^R_n$ and $d_n^R$. Because all coefficient vectors $\varvec{c}_{n}$ are assumed to vanish for $n>\vert n_\mathrm{max}\vert$ it follows that

109 $$\begin{aligned} \hat{S}_{n_\text {max}} = 0, \qquad \quad \hat{S}^R_{-n_\text {max}} = 0,\end{aligned}$$

110 $$\begin{aligned} \varvec{d}_{n_\text {max}} = 0, \qquad \quad \varvec{d}_{-n_\text {max}}^R = 0. \end{aligned}$$

This defines the initial condition that is needed to determine the remaining transition matrices and vectors for $0< n < n_\mathrm{max}$:

111 $$\begin{aligned} \hat{S}_{n-1}&= [\hat{K}_n -\hat{S}_n ]^{-1},\end{aligned}$$

112 $$\begin{aligned} \varvec{d}_{n-1}&= \hat{S}_{n-1} ( \varvec{d}_n + \varvec{\tilde{c}}_{n} ), \end{aligned}$$

and for $$-n_\mathrm{max}< n < 0$$

113 $$\begin{aligned} \hat{S}^R_{n+1}&= [\hat{K}_n -\hat{S}_n^R ]^{-1},\end{aligned}$$

114 $$\begin{aligned} \varvec{d}_{n+1}^R&= \hat{S}_{n+1}^R ( \varvec{d}^R_n + \varvec{\tilde{c}}_{n} ). \end{aligned}$$

To summarize, the rate response functions $r_{\ell , k}$ and hence the full firing rate can be calculated following an iterative scheme illustrated in Fig. 16. Starting point is the zeroth order term in the signal amplitude ($\ell =0$), where $\varvec{c}'_n = 0$ is known. For each iteration step, i.e. $\ell \rightarrow \ell +1$, the following series of steps is executed.

1. Compute the sum $\varvec{c}_n' := \varvec{c}_n^{(\ell -1,k+1)} + \varvec{c}_n^{(\ell -1,k-1)}$ of the *previously* computed coefficient vectors and the matrices involved in the computations of $K_n$ according to Eq. (99).
2. Compute all transition matrices $\hat{S}_n$ and vectors $\varvec{d}_n$ (and $\hat{S}_n^R$, $\varvec{d}_n^R$) iteratively starting at $n=n_\mathrm{max}$ (and $n=-n_\mathrm{max}$) using Eqs. (111)–(114).
3. Find all coefficient vectors $\varvec{c}_n := \varvec{c}_n^{(\ell ,k)}$ iteratively according to Eqs. (104) and (105) using the initial condition Eqs. (103) for $n=0$ and the transition matrices and vectors from the previous step.
4. Substitute the coefficients $c_{n,p}^{(\ell , k)}$ into Eq. (95) and determine the response functions $r_{\ell , k}$.

## C. MCF method: response to two periodic signals

In Sect. 4.3 we were interested in the nonlinear response *r*(*t*) of the noisy theta neuron subject to *two* periodic signals. To this end, we need to compute the response functions $r_{k_1,k_2}^{\ell _1,\ell _2}$ that are related to the expansion functions $P_{k_1,k_2}^{\ell _1,\ell _2}$ by Eq. (69). The latter in turn obey the following differential equation.

115 $$\begin{array}{l} \left( \hat{L}_0 +i \left( k_1 \omega _1 +k_2 \omega _2 \right) \right) P_{k_1,k_2}^{\ell _1,\ell _2} = \\\tfrac{\hat{L}_\text {per}}{2} \Big (P_{k_1+1,k_2}^{\ell _1-1,\ell _2} + P_{k_1-1,k_2}^{\ell _1-1,\ell _2}+P_{k_1,k_2+1}^{\ell _1,\ell _2-1}+P_{k_1,k_2-1}^{\ell _1,\ell _2-1} \Big ) \end{array}$$

This system of coupled differential equations can be solved iteratively as described in Sect. 4.3. We wish to find the function $P_{k_1,k_2}^{\ell _1,\ell _2}$ given that the functions on the right-hand side of Eq. (115) are already computed. For each $P_{k_1,k_2}^{\ell _1,\ell _2}$ the differential equation has in principle the same structure as Eq. (93) from the previous section. Hence, it can be solved using the same numerical scheme based on the MCF method, using Eqs. (94)–(114), except for a change in the notation that reflects the expansion with respect to *two* signals:

$$\begin{aligned} P_{\ell ,k}&\rightarrow P_{l_1,l_2}^{k_1,k_2},\\ r_{\ell ,k}&\rightarrow r_{l_1,l_2}^{k_1,k_2},\\ \varvec{c}_{n}^{(\ell ,k)}&\rightarrow \varvec{c}_{n}^{(\ell _1,\ell _2,k_1,k_2)}. \end{aligned}$$

Further two differences are:

1. Replace $k \omega$ by $k_1 \omega _1 + k_2 \omega _2$ which effects the computation of $K_n$ according to Eq. (99).
2. The function $P_{k_1,k_2}^{\ell _1,\ell _2}$ is determined by four previously computed expansion functions $P_{k_1+1,k_2}^{\ell _1-1,\ell _2}$, $P_{k_1-1,k_2}^{\ell _1-1,\ell _2}$, $P_{k_1,k_2+1}^{\ell _1,\ell _2-1}$ and $P_{k_1,k_2-1}^{\ell _1,\ell _2-1}$. This affects the computation of $\varvec{c}_n'$ as follows:
  116 $$\begin{aligned} \begin{aligned} \varvec{c}_n'&= \varvec{c}_{n}^{(\ell _1-1,\ell _2,k_1-1,k_2)} + \varvec{c}_{n}^{(\ell _1-1,\ell _2,k_1-1,k_2)}\\ {}&+\varvec{c}_{n}^{(\ell _1,\ell _2-1,k_1,k_2-1)} + \varvec{c}_{n}^{(\ell _1,\ell _2-1,k_1,k_2+1)} \end{aligned} \end{aligned}$$

Note that the known initial coefficient vector in Eq. (103) is still $\varvec{c}_0 = (1,0,...,0)^T$, when computing the stationary firing rate $r_{0,0}^{0,0}$ and $\varvec{c}_0 = (0,0,...,0)^T$ else.

The hierarchy of coupled differential equations is indeed different to the previous section and is provided up to the 2nd order in the signal amplitude in Sect. 4.3.

## Footnotes

1 One way to derive the QIF model is to consider the limit of a large slope factor $\Delta _v$ in the exponential integrate-and-fire model $C \dot{v} = I_0 - g_L v + g_L \Delta _v\exp ((v-v_t)/\Delta _v)$ which itself results from a simplification of a conductance-based model (Fourcaud-Trocmé et al., 2003). By choosing the new variable $x = (v-v_t)/(\sqrt{2}\Delta _v)$ and expanding the exponential function up to the second order for $v - v_t \ll \Delta _v$, one finds $\sqrt{2}\tau _m \dot{x} = \mu + x^2$, where $\tau _m = C/g_L$ is the membrane-time constant. For simplicity we neglected the prefactor $\sqrt{2}$ in Eq. (1).

2 How fast the MCF method converges with the number of Hermite functions and Fourier modes considered depends on the system parameters as demonstrated in the repository. More precisely, for a fixed $p_\text {max}$ or $n_\text {max}$ we observed that the MCF method fails for large correlation times and additionally in the excitable regime for small noise intensities. However, for these particular limit cases analytical approximations already exist (see Sects. 3.2 and 3.3). Choosing a $p_\text {max} = n_\text {max} \ge 150$, we can even capture these limit cases sufficiently well (see Fig. 3).
